# Molecular and cellular mechanisms of neutral lipid accumulation in diatom following nitrogen deprivation

**DOI:** 10.1186/1754-6834-6-67

**Published:** 2013-05-04

**Authors:** Zhi-Kai Yang, Ying-Fang Niu, Yu-Han Ma, Jiao Xue, Meng-Han Zhang, Wei-Dong Yang, Jie-Sheng Liu, Song-Hui Lu, Yuanfang Guan, Hong-Ye Li

**Affiliations:** 1 Key Laboratory of Eutrophication and Prevention of Red Tide of Guangdong Higher Education Institute, College of Life Science, Jinan University, Guangzhou, 510632, China; 2 Department of Computational Medicine and Bioinformatics, University of Michigan, Ann Arbor, Michigan, 48109, United States of America

**Keywords:** Microalga, Nitrogen deprivation, Lipid, Membrane remodeling, Transcriptomics

## Abstract

**Background:**

Nitrogen limitation can induce neutral lipid accumulation in microalgae, as well as inhibiting their growth. Therefore, to obtain cultures with both high biomass and high lipid contents, and explore the lipid accumulation mechanisms, we implemented nitrogen deprivation in a model diatom *Phaeodactylum tricornutum* at late exponential phase.

**Results:**

Neutral lipid contents per cell subsequently increased 2.4-fold, both the number and total volume of oil bodies increased markedly, and cell density rose slightly. Transcriptional profile analyzed by RNA-Seq showed that expression levels of 1213 genes (including key carbon fixation, TCA cycle, glycerolipid metabolism and nitrogen assimilation genes) increased, with a false discovery rate cut-off of 0.001, under N deprivation. However, most light harvesting complex genes were down-regulated, extensive degradation of chloroplast membranes was observed under an electron microscope, and photosynthetic efficiency declined. Further identification of lipid classes showed that levels of MGDG and DGDG, the main lipid components of chloroplast membranes, dramatically decreased and triacylglycerol (TAG) levels significantly rose, indicating that intracellular membrane remodeling substantially contributed to the neutral lipid accumulation.

**Conclusions:**

Our findings shed light on the molecular mechanisms of neutral lipid accumulation and the key genes involved in lipid metabolism in diatoms. They also provide indications of possible strategies for improving microalgal biodiesel production.

## Background

Concerns about global climate change and rises in prices of fossil fuels have prompted intense interest in the ability of microalgae to produce lipids that can be easily converted to biodiesel. Diatoms account for up to 40% of primary productivity in marine ecosystems [1] and some species are known to accumulate neutral lipids. Moreover, diatoms are metabolically versatile since they can synthesize and accumulate wide ranges of valuable compounds, such as polyunsaturated fatty acids (PUFAs), extracellular polymeric substances and cell coatings [2]. Hence, they have attracted both biological and medical attention. Recently, the marine diatom *Phaeodactylum tricornutum* has emerged as a potential microalgal energy source. It grows rapidly, has a short life cycle, and accumulates TAGs in late exponential phase; storage lipids constitute about 20-30% of its dry cell weight under standard culture conditions [3]. Furthermore, it is a model diatom species and its genome has been fully sequenced and available at the JGI (http://genome.jgi-psf.org/Phatr2/Phatr2.home.html) [4].

The accumulation of neutral lipids and changes in lipid profiles under nutrient starvation have been monitored in *P. tricornutum* and another model diatom, *Thalassiosira pseudonana*[5-7]. Nitrogen is a major constituent of proteins and nucleic acids. Accordingly, pigment and protein losses have been observed in Antarctic sea ice diatoms under nitrogen limitation [8], and while N-starved *P. tricornutum* cells can accumulate high levels of lipids, their division reportedly ceases and cell density increases only marginally [1,9,10]. Furthermore, changes in fatty acid profiles of the green alga *Chlamydomonas reinhardtii* under N deprivation have been reported recently [11], and associated shifts in fluxes through metabolic pathways have been inferred from changes in transcript abundance in *C. reinhardtii*[12]. However, the biochemical and molecular mechanisms involved in diatom responses to losses of nitrogen availability are still unclear. Therefore, in the study reported here *P. tricornutum* was cultured under standard laboratory conditions then subjected to nitrogen deprivation in the late exponential phase to achieve both high neutral lipid contents and high cell biomass. Then, to assess the holistic effects of N deprivation and identify the mechanism involved in the diatoms, we examined changes in their levels of neutral lipids, oil bodies, fatty acid profiles, chloroplast structure, photosynthetic parameters, gene expression patterns and inferred shifts in fluxes through key metabolic pathways.

## Results and discussion

### Effects of N deprivation in late exponential growth phase on growth and neutral lipid accumulation in *P. tricornutum*

**Figure 1:**
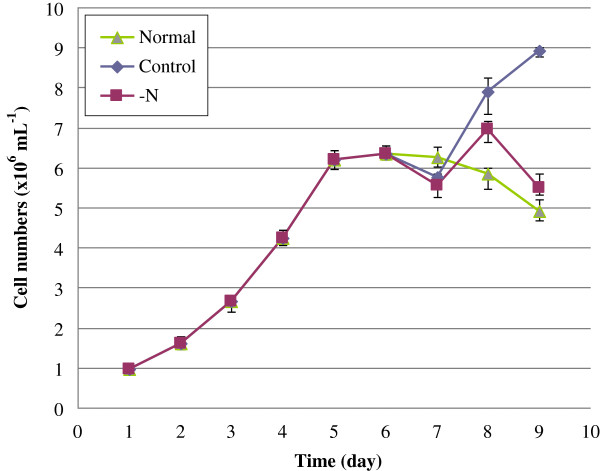
**Growth of *P. tricornutum *under standard and N-free conditions.** Normal, cells grown under standard conditions, i.e. continuous culture in f/2-Si medium; Control, cells cultured in f/2-Si medium, with a transfer to fresh medium 5 days after subculture; -N, cells cultured in f/2-Si medium, with a transfer to fresh N-free medium 5 days after subculture.

To monitor their growth, the *P. tricornutum* cultures were sampled at the same time (14:00) daily and cells in the samples were counted using a hemacytometer. As shown in Figure 1, the cultures grown solely under standard conditions showed typical growth curves. Switching to fresh medium (controls) in late exponential phase stimulated growth, and switching to N-free medium (N deprivation, -N) induced an intermediate response; slightly lower growth than in the controls, but higher than that of the standard continuous cultures. These findings indicate that N deprivation in late exponential phase can stimulate further increases in cell biomass, and intracellular N reserves can be used by diatom cells to fuel growth even if the extracellular N concentration is low. It should be noted that the cells showed typical growth characteristics in the absence of silicon, in accordance with findings by De Martino *et al*. [13]. Thus, silicon was omitted from the culture medium used in further experiments, to prevent the formation of frustules and thus facilitate cell breakage.

**Figure 2:**
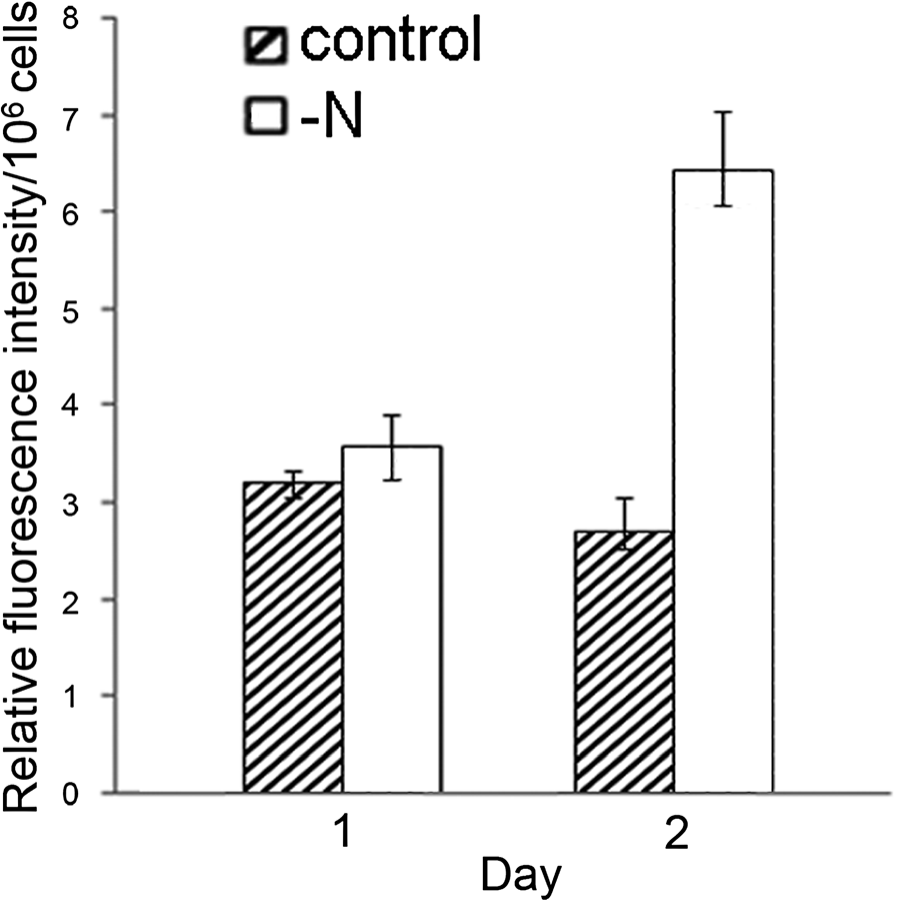
**Relative fluorescence intensity of diatom cells under N deprivation.** Triplicate samples of diatom cells were stained with Nile Red after 1 and 2 days of N deprivation, their fluorescence was measured and their relative fluorescence intensity was calculated by subtracting the autofluorescence of non-stained microalgae and Nile red. The left and right columns in each day reprensent the control and –N, respectively.

The lipid contents of the cultures were assessed, rapidly and conveniently, using Nile Red and a fluorimetric spectrophotometer with a 480 nm excitation wavelength. The optimal emission and staining time were empirically found to be 592 nm and 20 min, respectively, similar to previously optima for neutral lipid determination: 480 nm and 580–610 nm emission and excitation wavelengths, respectively, with 10-30 min staining time [14]. The fluorescence intensity per cell was significantly (10%) higher than that of the controls after one day of N deprivation, and 2.4-fold higher after two days (Figure 2), which was basically in line with the lipid contents of 24.13% (control) and 53.33% (−N) obtained after –N for 2 days by the gravimetric determination (Table 1). These results confirmed that N-deprivation induces substantial neutral lipid accumulation and that *P. tricornutum* is a promising biodiesel resource. However, the growth rate of the N-deprived cultures was slightly lower than control rates following the medium changes, although higher than that of the cultures kept in the same medium throughout the experiment. The findings indicate that a two-step culture strategy with N deprivation could deliver much higher neutral lipid yields than strategies based simply on continuous N deficiency [15].

**Table 1:** **Fatty acid composition of *P. tricornutum *following N deprivation**

**Fatty acids**	**Control**		**-N**	
	**Total FA%**	**DW%**	**Total FA%**	**DW%**
C14:0 (myristic)	5.31	0.22	6.27	0.12
C16:0 (palmitic)	15.89	0.66	25.87	0.49
C18:0 (stearic)	2.67	0.11	2.37	0.04
Sum SFA	23.87	0.99	34.51	0.65
C16:1 (palmitoleic)	23.91	0.99	42.55	0.80
C18:1 (oleic)	2.45	0.10	NT	ND
Sum MUFA	26.36	1.09	42.55	0.80
C18:2 (linoleic)	NT	NT	5.22	0.10
C18:3 (linolenic)	2.69	0.11	1.25	0.02
C20:5 (eicosapentaenoic)	20.33	0.84	11.67	0.22
C22:6 (docosahexaenoic)	1.07	0.04	0.64	0.01
Sum PUFA	24.09	1.00	18.78	0.35
other FA	10.88	0.45	2.71	0.05
Total FA identified	85.20	3.53	98.55	1.85
Unsaturation ratios:				
C 16 unsat./C16:0	1.50	0.06	1.64	0.03
C18 unsat./C18.0	1.93	0.08	2.73	0.05
Total unsat./total sat.	2.11	0.09	1.78	0.03
Lipid content (DW)		24.13		53.33

Abbreviations: *FA*, Fatty acid; *DW*, Dry weight; *SFA*, Saturated fatty acid; *MUFA*, Monounsaturated fatty acid; *PUFA*, Polyunsaturated fatty acid; *ND*, not detected.

### Changes in fatty acid composition under N deprivation

As shown in Table 1, substantial differences in fatty acid composition between N-deprived and control cells were detected by GC-MS analysis: proportions of saturated fatty acids (SFA) and monounsaturated fatty acids (MUFA) were 45% and 61% higher in the former, respectively, while the proportion of polyunsaturated fatty acids (PUFA) was 22% lower. The proportions of other fatty acids also markedly declined in the N-deprived cultures. The total unsaturated lipid content decreased by 16% under N deprivation, and proportions of the long-chain fatty acids C20:5 and C22:6 were 43% and 40% lower, respectively. The changes in fatty acid composition were largely due to dramatic increases in palmitic and palmitoleic acids, with accompanying losses of eicosapentaenoic acid, which is generally one of the most abundant fatty acids in *P. tricornutum*[16].

### Observation of oil bodies by confocal microscopy

**Figure 3:**
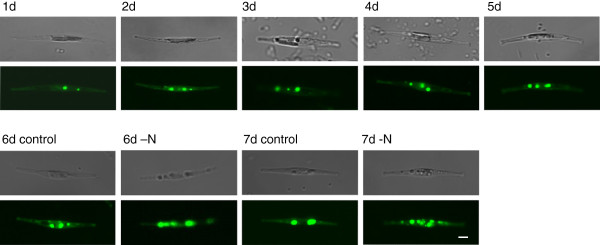
**Representative confocal microscope images of the diatoms, showing oil bodies.** Cells sampled during a 7-day culture cycle were stained with Nile Red and photographed under the confocal microscope daily. –N, cells subjected to the N deprivation treatment five days after subculture; Control, cells grown in f/2-Si medium and switched to fresh medium five days after subculture. Bar = 2 μm.

The size and number of oil bodies in sampled cells were observed every day during the culture cycle using a laser scanning confocal microscope. Substantially more oil bodies were observed in the N-deprived cells than in controls, but their sizes were similar (Figure 3), with diameters ranging from 0.2 to 2.0 μm. Thus, the total volume of oil bodies was clearly higher in the N-deprived cultures than in the controls, in accordance with the neutral lipid increase, as shown in Figure 2.

### High-throughput analysis of differential gene expression

To characterize the effects of N deprivation further, we examined differences in gene expression before and after N-deprivation, using RNA-Seq. The complete dataset is listed in Additional file 1, and also available at http://guanlab.ccmb.med.umich.edu/data/Yang_N_deprivation/, but briefly 1213 genes were found to be up-regulated, with an FDR cutoff of 0.1%, and 4527 genes were down-regulated, indicating that the deprivation induced wide re-programming of regulation. Distribution of the number of reads across different mRNA sequence size indicates a significant correlation (Additional file 2: Figure S1). To validate the findings we applied quantitative RT-PCR (qPCR) to a randomly selected set of genes, including β-actin as a housekeeping marker, to test the differential expression. The RNA-Seq measurements are highly consistent with the quantitative PCR results, confirming their robustness. For example, Phatrdraft_27877, encoding an ammonium transporter protein, was up-regulated 5.46 (log_2_)-fold and 116-fold under N-deprivation, according to the RNA-Seq and qPCR analyses, respectively (Table 2).

**Table 2:** qPCR of differentially expressed genes following N deprivation

**qPCR**				**RNA-Seq**		
**Locus tag**	**Forward (5’-3’)**	**Reverse (5’-3’)**	**FC(N/Ctr)**	**FC Log**_**2**_**(N/Ctr)**	**Z-test**	**FDR**
**54257**	agaatcggtgatgcctgttc	atgccgctgctttagtgaat	103.3	5.80	0	0
**45012**	acgattcggacgaagatcag	ccatgcaacaatcgtagtgg	26.0	5.56	6.71E-14	1.99E-13
**27877**	acaccaccgacaagaccttc	tccagacagagcgtacaacg	116.6	5.46	0	0
**54465**	gagcacttcgttctccaagg	gtccagaaagccacagcttc	−17.1	−8.98	0	0
**13154**	caggaactcgcgaagttagg	gcaagaatggaacccactgt	3.35	7.42	2.46E-5	4.37E-5
**45852**	aaggccacaatctcatggac	cttttgacggatggcaactt	23.6	7.41	7.47E-9	1.63E-8
**51183**	ctttacaacgccctgatcgt	ttgctgtcgtggaaagactg	−15.7	−8.65	0	0
**9794**	gcatattggaggctttggaa	tctgcatcatcatcccgata	1.35	−0.58	7.29E-3	0.010

Each value corresponds to the mean of three biological sample performed in duplicates; β- actin is used as house-keeping gene in real-time quantitative PCR; significant changes are indicated by a *p* value<0.05 (Z-test).

### General transcriptional changes under N deprivation

**Figure 4:**
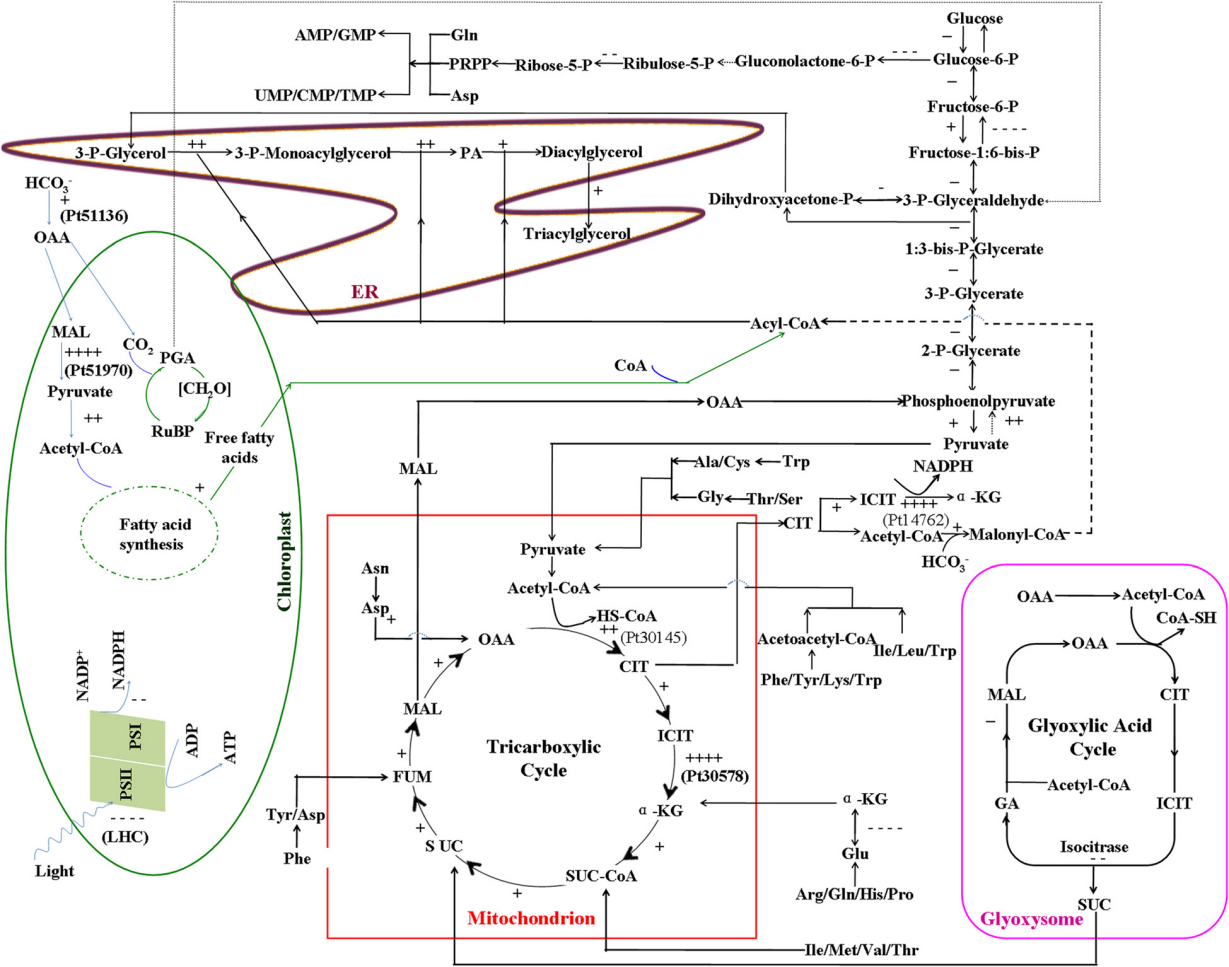
**Proposed general transcriptional changes under N deprivation.** Schematic diagram showing the putative localization of central metabolic pathways of *P. tricornutum*. Changes in the transcript abundance of associated genes are indicated with the following symbols representing fold changes: ++++ > 5; 5 > +++ > 3; 3 > ++ > 1; 1 > + > 0; 0 < − < −1; -1 < −−− < −3; -3 < −−− < −5; −−− < −5. The symbol denotes the overall changes of predicted isoenzymes. Some co-substrates or co-products of some reactions are omitted for clarity. The one “+” in the fatty acid synthesis in plastids denotes the overall transcriptional changes of the enzymes involved. Detailed fold changes are listed in Additional file 3 and Additional file 1. Enzymes were based on the genome annotation and prediction. Locus tags of some ambiguous isoenzymes are indicated. The localization of most of the enzymes are based on prediction and have not been determined experimentally.

To elucidate the molecular basis of the observed accumulation of fatty acids under N deprivation, genes encoding all known enzymes in the *P. tricornutum* genome were mapped in KEGG pathways, together with log_2_-fold differences in RNA expression between the control and N-deprived cultures. Global changes in major categories of genes involved in various pathways, reflecting general transcriptional responses to N deprivation, are depicted in Figure 4 and listed in Additional file 3: Table S1a. Genes encoding photosynthesis, gluconeogenesis, glyoxylate cycle, chrysolaminarin synthesis and sucrose metabolism enzymes were, on average, significantly down-regulated under N deprivation, while genes involved in nitrogen fixation, carbon fixation, glycolysis and the TCA cycle were generally up-regulated.

The primary effect of N deprivation is the reduced availability of nitrogen, an essential nutrient for *de novo* synthesis of amino acids, nucleic acids and various other cellular constituents. Unsurprisingly, therefore, it strongly affected transcript levels of genes involved in nitrogen assimilation and metabolism (Additional file 3: Table S1b). Notably, marked increases were observed in transcripts encoding five of at least six ammonium transporters present in the diatom, which transport ammonium ions across the cell membrane and are known to be activated by N deprivation [17]. The rise in the level of one of them was also confirmed in our qPCR test (Phatrdraft_27877, 45-fold increase). In addition, there was a 7-fold increase in transcripts of glutamine synthase (GS, Phatrdraft_22357, EC 6.3.1.2), a key enzyme in ammonia assimilation in both plants and Gram-negative microorganisms. GS plays an important role in the efficient use of nitrogen sources and nitrogen metabolism. Other critical nitrogen metabolism enzymes, such as nitrate reductase (Phatrdraft_54983, EC1.7.7.2), a molybdoenzyme that reduces nitrate to nitrite, and ferredoxin-nitrite reductase (Phatrdraft_12902, EC1.7.7.1), which catalyzes conversion of nitrite to ammonia, also showed significant increases (4-fold and 33-fold, respectively) in transcript abundance. These observations indicate that N deprivation induces homeostatic responses, including activation of the glutamine synthesis pathway and increases in the cells’ capacity to utilize trace amounts of nitrogen resources, and also the possible redistribution of intracellular nitrogen such as increased amino acid catabolism in accordance with the study in *T. pseudonana*[6], ultimately yielding acetyl-CoA or succinyl-CoA as the TCA cycle intermediates.

### Transcripts of photosynthesis-related genes decreased

As shown in Additional file 3: Table S1c, levels of most transcripts encoding proteins associated with photosynthesis decreased under N deprivation, implicating that photosynthesis could be inhibited or at a standstill. Notably, only one encoding a light harvesting complex (LHC) component was up-regulated (Phatrdraft_16481, fucoxanthin chlorophyll a/c protein; up-regulated 10-fold), while most of the others were down-regulated more than 10-fold. As previously demonstrated in *C. reinhardtii*, reductions in the abundance of light-harvesting complexes may reduce photosynthetic rates [18], in accordance with theoretical expectations, unless reductions in transcript levels are balanced by changes at other levels. Transcript levels of ferredoxin-NADP^+^ reductase (Phatrdraft_43018), which catalyzes the last electron transfer, from photosystem I to NADPH during photosynthesis [19], also decreased (4.6-fold).

The reductions in mRNA levels of genes encoding photosynthetic proteins under N deprivation were consistent with expectations since various stresses can inhibit photosynthesis, and upregulated light harvesting can result in excess production of highly toxic reactive oxygen species (ROS) [20]. To examine whether the reduced expression of photosynthetic genes was reflected in physiological changes, we also measured and calculated chlorophyll fluorescence parameters of the cultures, including Fo, Fm, Fv/Fm and Fv/Fo. As shown in Table 3, all of these parameters significantly decreased under N deprivation, indicating that the stress impaired PSII reaction centers, reduced the chlorophyll fluorescence yield, and decreased both the primary light energy conversion efficiency and potential photochemical activity of the PSII reaction centers [21]. The inhibition of genes associated with photosynthesis by N deprivation observed in this study is consistent with reported responses of *P. tricornutum*[7] and green alga *Chlamydomonas*[12,22], and can be considered a generic response to nutrient deficiencies. A practical implication of the findings for industrial biodiesel production is that light may not be required for neutral lipid accumulation under N deprivation. However, a previous report indicates that polysaccharides are produced at high rates during light periods and consumed in the dark, while the total lipid contribution to dry weight is positively associated with light intensity in N-deficient *P. tricornutum*[9]. Thus, the effects of light on lipid accumulation in diatoms, and its interactive effects with other environmental factors, clearly require further characterization.

**Table 3:** Measurement of photosynthetic activity

**Group**	**Chlorophyll fluorescence parameters**			
	**Fv/Fm**	**Fv/Fo**	**Fo**	**Fm**
**Control**	**0.604 ± 0.003**	**1.520 ± 0.019**	**323.75 ± 2.50**	**816.75 ± 7.23**
**-N**	0.550 ± 0.0164**	1.221 ± 0.080**	184.25 ± 4.86**	409.00 ± 5.42**

Note: Chlorophyll fluorescence parameters were monitored 48 h post N deprivation (−N). Date are shown as means $\bar{\times }$ ±SD; * and ** denote significant difference (*p* < 0.05) and extremely significant difference (*p* < 0.01), respectively.

### Increased carbon assimilation and carbon fluxes towards TAG accumulation

Two genes encoding putative isoforms of phosphoenolpyruvate carboxylase (PEPC, EC4.1.1.31), Phatrdraft_27976 and Phatrdraft_51136, are present in the *P. tricornutum* draft genome and both were up-regulated moderately under N deprivation, 1.5-fold and 2-fold, respectively (Additional file 3: Table S1d). The two PEPCs were predicted to be localized in the chloroplast (Phatrdraft_27976; mito: 7.0, chlo: 7.0; Phatrdraft_51136, chlo: 9.0, cyto: 5.0, by WoLF PSORT) implicating the presence of C4 photosynthetic pathway. Two green microalgae, *C. reinhardtii* and *Selenastrum minutum*, also reportedly possess at least two distinct PEPC isoforms, which differ significantly from their plant and prokaryotic counterparts [23]. PEPC catalyzes the irreversible β-carboxylation of phosphoenolpyruvate (PEP) in the presence of HCO_3_- and Me^2+^ to yield inorganic phosphate and oxaloacetate (OAA). Thus, it is intimately involved in C_4_-dicarboxylic acid metabolism in these organisms. It also plays an anaplerotic role in most non-photosynthetic organs of plants, C_3_ leaves and microalgae (including diatoms), providing OAA and/or malate to replenish citric acid cycle intermediates consumed in other primary metabolic pathways, notably ammonia assimilation [24]. PEPC is considered to be a key enzyme for carbon fixation in diatoms [18,24]. In fact, the moderate up-regulation of the two PEPCs here was basically in accord with the other reports that *P. tricornutum* PEPC transcripts showed no significant increase under low CO_2_ conditions [7,25]. Although the carbon fixation pathway in *P. tricornutum* has not been fully elucidated, the deduced pathway in some diatoms [18,25-27] and increases in transcript levels of both PEPCs observed here suggest that it can take up inorganic carbon and possesses a CO_2_ concentration mechanism (CCM) (Additional file 4: Figure S2).

In addition, transcript levels of a predicted malic enzyme (NADP^+^-dependent ME; EC1.1.1.40, Phatrdraft_51970), a component of the putative carbon fixation pathway, with a predicted chloroplast localization (chlo: 8.0, cyto: 6.0, by WoLF PSORT), dramatically increased, over 7-fold (Additional file 4: Figure S2; Additional file 3: Table S1d). ME catalyzes a rate-limiting step for fatty acid biosynthesis; the irreversible decarboxylation of malate to pyruvate with the formation of NADPH from NADP^+^, hence providing essential reducing power for fatty acid biosynthesis via fatty acid synthase. Another previously reported putative decarboxylating enzyme of the malic enzyme family encoded by Phatrdraft_56501 [27], was predicted to be mainly chloroplast location (chlo: 11.0, mito: 2.0, by WoLF PSORT). Its presence is also consistent with reports of a single-cell C_4_ pathway in marine diatoms, including *P. tricornutum*, involving OAA entering the chloroplast [25]. Wynn *et al.* have investigated the role of ME in filamentous fungi [28] and found that its overexpression increases NADPH production, thereby providing reducing power and cofactors for reactions catalyzed by enzymes involved in TAG synthesis such as ACCase, fatty acid synthase (FAS), ultimately leading to increases in TAG accumulation. Similarly, overexpression of two ME genes from *Mucor circinelloides* and *Mortierella alpine* in *Mucor circinelloides* reportedly increased ME activity 2- to 3-fold, and increased lipid contents from 12% to 30% of total biomass [29]. Therefore, the dramatic increase in ME transcription we observed under N deprivation could contribute substantially to the accumulation of neutral lipids in *P. tricornutum*. In contrast, levels of pyruvate-phosphate dikinase (EC2.7.9.1, Phatrdraft_21988) transcripts decreased 7.5-fold. Pyruvate-phosphate dikinase is a key enzyme in the C_4_ photosynthetic pathway [30], thus reduction in its expression could reduce photosynthesis rates. Under such circumstances, increases in PEPC expression or activities in *P. tricornutum* could optimize use of the available phosphoenolpyruvate for carbon fixation, while saving most pyruvate for acetyl-CoA formation. Further, decreases in pyruvate-phosphate dikinase activity may prevent possible activation of alternative gluconeogenic pathways, such as that reportedly observed in *Rhizobium (Sinorhizobium) meliloti*[30]. Taken together, the transcriptional changes in carbon assimilation under N deprivation may substantially increase carbon influxes, providing a rich source of substrate for fatty acid production. In fact, Fourier transform infrared spectrometer (FTIR) assay conducted according to Jiang *et al*. [31] showed a slight increase of carbohydrate under N deprivation (data not shown), which is basically in line with the previous findings in *P. tricornutum*[7] and *T. pseudonana*[31]. Furthermore, the 2-fold increase of C:N ratio during N depletion according to Valenzuela et al. [7] together with the slight increase of carbohydrate implicate that the carbon is challenged into the production of storage lipid during -N.

*P. tricornutum* appears to possess at least five isoforms of fructose-1,6-bisphosphatase (FBP, EC3.1.3.11), a key regulatory enzyme of carbon metabolism (particularly the Calvin cycle and gluconeogenesis), which catalyzes the conversion of fructose-1,6-biphosphate to fructose-6-phosphate (Additional file 3: Table S1d). Transcript levels of genes encoding four of these isoforms, Phatrdraft_2793, Phatrdraft_9359, Phatrdraft_31994 and Phatrdraft_8744, declined under N deprivation, 45-, 17-, 3- and 3.5-fold, respectively, while those of the other (Phatrdraft_23247) slightly increased, by 50%. Therefore, gluconeogenesis was markedly inhibited and carbon flux was re-directed towards TAG accumulation under N deprivation. In contrast, two genes encoding phosphofructokinase (EC2.7.1.11), which catalyzes the committed step in the glycolytic pathway (conversion of fructose-6-phosphate to fructose-1,6-bisphosphate) were up-regulated: Phatrdraft_55126 and Phatrdraft_16844, by 36% and 50%, respectively. The up-regulation of glycolysis should theoretically direct carbon flux to the formation of pyruvate. Moreover, levels of transcripts encoding a precursor of the pyruvate dehydrogenase E1 component beta subunit (Phatrdraft_20183, EC1.2.4.1) and the alpha subunit (Phatrdraft_55035) increased 3-fold and slightly (by 15%), respectively. These findings propose that conversion of pyruvate to acetyl-CoA may be activated under N deprivation, thereby providing substrate for the citric acid (TCA) cycle following the conversion of acetyl-CoA to citrate as well as building up precursors to ACCase for fatty acid biosynthesis. And we found that the predicted cytosolic isocitrate dehydrogenase (Phatrdraft_14762) increased 16-fold, which is involved in the conversion of citrate to α-ketoglutarate. During this process, NADP^+^ is reduced to NADPH which is a critical cofactor for many enzymatic reactions in lipid biosynthesis [32].

Diatoms do not possess starch-forming enzymes, but store fixed carbon as a complex, soluble carbohydrate called chrysolaminarin (β-1, 3-glucan) in vacuoles. Transcript levels of phosphoglucomutase (Phatrdraft_50445), which catalyzes the rate-limiting step of carbohydrate synthesis, decreased 3.7-fold under N deprivation. This is consistent with both the observations of neutral lipid accumulation and recent reports that N-deprived *C. reinhardtii* mutants with phosphoglucomutase deficiencies store neutral lipids in lipid bodies [33,34]. Furthermore, since chrysolaminarin and TAG syntheses share common 3-carbon photosynthate precursors, mutations or reductions in the expression of phosphoglucomutase could affect chrysolaminarin biosynthesis and redirect carbon fluxes to TAG accumulation. The decrease in carbohydrate contents under N deprivation is consistent with previous findings that high lipid contents in *P. tricornutum* are accompanied by low total carbohydrate contents [9].

Almost all of the genes involved in the TCA cycle—which converts 2-oxoglutarate, coenzyme A and NAD(+) to succinyl-CoA, NADH and carbon dioxide—were activated under N deprivation (Additional file 4: Figure S2; Additional file 3: Table S1e). Levels of transcripts encoding two potentially rate-limiting enzymes of the TCA cycle—citrate synthase (Phatrdraft_30145, EC2.3.3.1) and isocitrate dehydrogenase (EC1.1.1.42, Phatrdraft_30578) increased 3- and 22-fold, respectively. In addition, those of a third TCA cycle enzyme, oxoglutarate dehydrogenase (Phatrdraft_29016, EC1.2.4.2), which converts 2-oxoglutarate to succinyl-CoA [19], slightly increased under N deprivation. These findings suggest that TCA cycle genes were markedly up-regulated under N deprivation, thus increases in fluxes through the TCA cycle may compensate for the loss of assimilatory power for carbon fixation due to the down-regulation of photosynthesis.

The transcript abundance of isocitrate lyase (Phatrdraft_14401, EC4.1.3.1) and malate synthase (Phatrdraft_54478), two glyoxylate cycle enzymes identified in the *P. tricornutum* genome, decreased 5- and 2.5-fold, respectively. This implies that the metabolic flux through the glyoxylate cycle could be reduced under N deprivation, thereby further inhibiting overproduction of oxaloacetate and gluconeogenesis. Similarly, transcripts encoding phosphoenolpyruvate carboxykinase (Phatrdraft_55018), which catalyzes the committed step of gluconeogenesis, decreased by 20%.

### Multiple sources for TAG accumulation and reductions in lipid catabolism

Transcript levels of genes associated with TAG biosynthesis were up-regulated by N-deprivation (Additional file 3: Table S1f). Notably, mRNA levels of Phatrdraft_9794, encoding diacylglycerol acyltransferase (DGAT, EC2.3.1.20), increased by 30%, according to both the RNA-Seq and qPCR analyses (Table 2). DGAT catalyzes the final committed step of TAG biosynthesis, thus the increase in its mRNA abundance under N deprivation may have increased TAG levels somewhat, but not as much as the observed increase in neutral lipid contents. Similarly, in *C. reinhardtii* there are two DGAT homologs, and transcripts encoding (DGTT2) are reportedly present at consistently low levels under all tested conditions, including N deprivation, while mRNA of the other (DGTT3) is present at low levels and only increases slightly under N deprivation [12]. The ability of increases in DGAT expression to raise TAG levels has been demonstrated by heterologous expression of *P. tricornutum* DGAT (PtDGAT1, Phatrdraft_9794) in a *Saccharomyces cerevisiae* neutral lipid-deficient quadruple mutant strain, which restored TAG and lipid body formation, and promoted incorporation of saturated fatty acids into TAGs [35]. Similarly, overexpression of *Tropaeolum majus* DGAT1 in *Brassica napus* and *Arabidopsis thaliana* resulted in 11–30% net increases in seed oil content [36].

Comparing with the much more increase of neutral lipid levels in *P. tricornutum* under N deprivation (2.4-fold), the slight increase of DGAT transcripts could be less effective. We examined further potential sources of substrates for TAG accumulation, and found that mRNA levels of another enzyme, responsible for the last step of TAG biosynthesis, phospholipid:diacylglycerol acyltransferase (PDAT, Phatrdraft_8860, EC2.3.1.158), increased 2-fold. Interestingly, in addition to the DGAT-catalyzed pathway, another acyl-CoA independent pathway catalyzed by PDAT for TAG synthesis has been discovered in *Arabidopsis*, in which acyl is directly transferred from phosphatidylcholine (PC) to DAG and thus TAG is synthesized without use of CoA as an intermediate [37]. Phosphatidate phosphatase (PAP, EC3.1.3.4, Phatrdraft_40261) mRNA levels increased by 25%, which were responsible for the intermediate DAG for PDAT-catalyzed TAG accumulation. These findings suggest that the PDAT-mediated pathway or intracellular membrane remodeling may have contributed to the observed TAG accumulation.

The transcript abundance of genes involved in fatty acid elongation decreased under N deprivation. Delta 6 elongase (Phatrdraft_20508) and long chain acyl-CoA elongase (Phatrdraft_34485) levels decreased by 16% and 32%, respectively, which is consistent with observed reductions in C20:5 and C22:6 fatty acids, as shown in Table 1. Responses of genes encoding fatty acid desaturases varied. Transcript abundance of two desaturases catalyzing PUFA formation (delta 12 fatty acid desaturase, Phatrdraft_25769, and delta 9 fatty acid desaturase, Phatrdraft_28797) decreased, by 2.6- and 4-fold, respectively. In contrast, the expression of delta 5 fatty acid desaturase (Phatrdraft_46830), involved in MUFA synthesis, increased dramatically (32-fold). These results are in line with the higher MUFA and lower PUFA proportions of fatty acids under N deprivation (Table 1).

In accordance with the general increases in transcript levels of genes involved in TAG synthesis, levels of those involved in lipid catabolism generally decreased under N deprivation, those of acyl-CoA oxidase (Phatrdraft_19979) and 3-oxoacyl-CoA thiolase (ATO1) most dramatically; 11-fold and more than 3-fold, respectively.

Lipases are enzymes that de-esterify carboxyl esters, such as TAGs and phospholipids. More than 28 genes encoding putative lipases are present in the *P. tricornutum* genome (Additional file 3: Table S1g) and their responses to N deprivation varied markedly. Since TAG accumulated markedly under N deprivation, TAG lipases would be expected to be down-regulated. Among the putative lipase genes, eight (28%) showed increased transcript abundance but mainly only slightly, and transcript levels of most of the others decreased more than 2-fold. For example: Phatrdraft_44231, which encodes a putative triacylglycerol lipase, decreased 5-fold; Phatrdraft_50397 decreased 4-fold; while 43593 increased nearly 4-fold. These lipase genes may play important roles in membrane turnover, and their overall reduction in transcription may protect the TAG from degradation, thereby promoting remodeling and TAG accumulation under N deprivation.

### Effects of N deprivation on the subcellular ultrastructure

**Figure 5:**
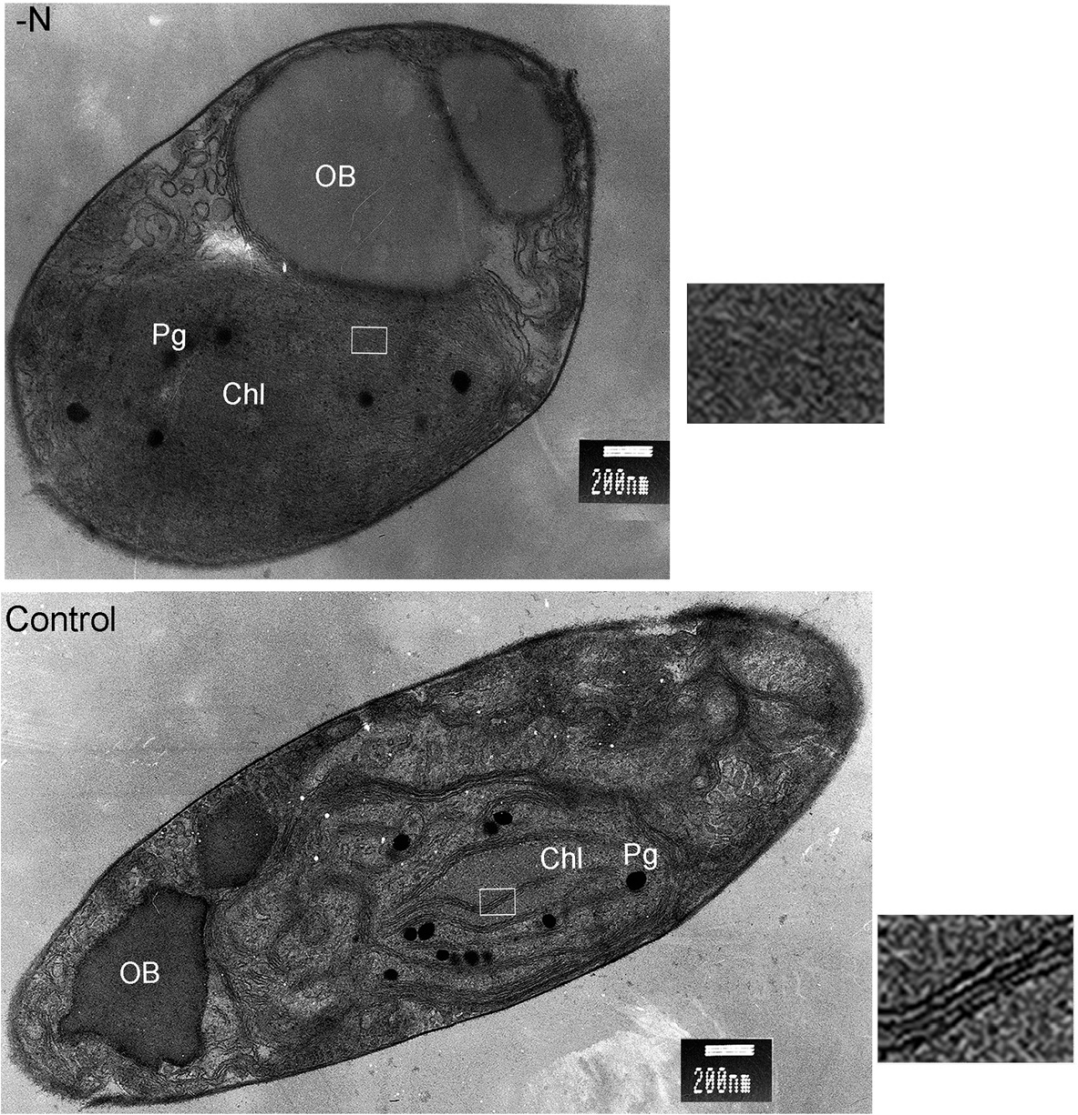
**Effects of N deprivation on *P. tricornutum *ultrastructure.** Transmission electron micrographs showing subcellular structures of N-deprived (−N) and control *P. tricornutum* cells. OB, oil body; Chl, chloroplast; Pg, plastoglobule; boxed areas are magnified and showed to the right, demonstrating the dispersed thylakoid membranes under -N. Bar = 200 nm.

Sections of cells were observed by transmission electron microscopy to assess the impact of N deprivation on their ultrastructure. Numerous oil bodies, of various sizes, were present in both the N-deprived and control cells. However, their total volumes were substantially higher in the former (Figure 5). The oil bodies in the N-deprived cells were globular, compact, and mostly 0.1 to 0.3 μm in diameter, but a few (2 to 5 per section) were much larger, up to 2.0 μm. In addition to causing significant changes in oil body contents, N deprivation strongly affected chloroplast ultrastructure. Diatoms are protists that contain typical secondary plastids surrounded by four membranes. Thus, they differ strongly from those of the algae *sensu stricto* (in particular, green algae such as *Chlamydomonas*) and vascular plants such as *Arabidopsis*, which contain simple plastids bounded by two membranes). In the control cells, although the cultures were in stationary phase, the chloroplast and thylakoid membranes were still highly organized. In contrast, the four enclosing membranes and thylakoid membrane system tended to be dispersed and poorly organized under N deprivation.

The main membrane lipids in plant chloroplasts are monogalactosyldiacylglycerol (MGDG) and digalactosyldiacylglycerol (DGDG). In *Arabidopsis*, these galactolipids are critical for the biogenesis of photosynthetic membranes, and provide sources of PUFA for the whole cell and phospholipid surrogates under phosphorus-limited conditions. Further, dramatic accumulations of DGDG accompanied by major reductions in membrane phospholipids have been observed in phosphorus-limited *Arabidopsis*, suggesting that the plant can substitute DGDG for phospholipids during phosphate starvation [38]. These galactolipids are synthesized by MGD and DGD galactosyl-transferases/synthases, which also mediate membrane lipid remodeling [38]. Three genes encoding putative monogalactosyldiacylglycerol synthases (EC 2.4.1.46) were detected in the *P. tricornutum* genome: Phatrdraft_9619, Phatrdraft_54168 and Phatrdraft_14125, which showed 6-, 6- and 9-fold decreases in transcript levels, respectively, under N deprivation. These results imply that membrane lipid remodeling, mediated by reductions in the expression of MGDG and/or DGDG galactosyltransferases, occurred in the N-deprived cells.

To further assess the contribution of membrane remodeling to lipid accumulation, total lipid extracts were separated into various lipid classes (Additional file 5: Figure S3) and analyzed by ESI/MS. The results showed that there were nine main classes of lipids in the *P. tricornutum* cells, and peak area measurements indicated that the proportions of MGDG and DGDG in the cells markedly decreased 50% under N deprivation, while the proportion of TAG (the major component of neutral lipids, mixed with photosynthetic pigments) appeared doubled.

We also analyzed the acquired transcription dataset to identify down-regulated genes that were annotated to the chloroplast. Out of 50 genes annotated to thylakoids, 45 were down-regulated, and only five were up-regulated. Our results demonstrate that lipid remodeling provides potential pathways for converting membrane lipids to substrates for TAG accumulation in *P. tricornutum*’s responses to N deprivation.

Moreover, electron-dense osmiophilic globuli previously reported as plastoglobuli [39] were detected in the stroma of both N-deprived and control cells, but there were fewer in the former (Figure 5). The functions of plastoglobuli and triggers for their formation are not well understood. However, protein profiles of plastoglobuli indicate that metabolic enzymes accumulate in them and they have distinct lipoprotein structures [39]. In addition, overexpressing the plastoglobule structural protein fibrillin in plants results in increased frequencies of plastoglobuli [40], suggesting that fibrillin is involved in their formation. Transcripts encoding fibrillins in *P. tricornutum* (Phatrdraft_48066 and Phatrdraft_55153, encoding PAP-fibrillin-1 and PAP-fibrillin-2) decreased 4- and 5-fold, respectively. This could at least partially explain the decrease of plastoglobuli in N-deprived cells.

## Conclusion

The overall goal of this study was to use genomic information and various analytical techniques to obtain an overview of the molecular mechanisms responsible for neutral lipid accumulation in the model diatom *P. tricornutum* induced by N deprivation. N-deprived cultures of the diatom showed up to 2.4-fold increases in neutral lipid contents per cell, slightly higher cell density than controls and exhibited ultrastructural changes. In addition, the lipid composition of the N-deprived cells was more suitable for biodiesel production as they had higher proportions of saturated fatty acids.

**Figure 6:**
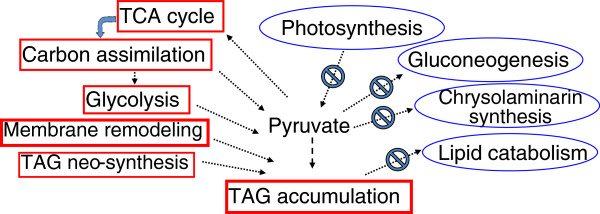
**Schematic diagram indicating effects of N deprivation on processes involved in TAG accumulation based on transcriptional changes.** Square and elliptical boxes indicate up-regulation and down-regulation, respectively. “ѳ” indicates inhibition of the corresponding process.

Although the level of transcription is not directly proportional to the enzyme and the catalytic activity, recent study on another diatom *T. pseudonana* has shown a good correlation of proteomic and transcriptomic data [6]. In combination with biochemical investigations, our results could provide a global view of biosynthetic metabolic fluxes and unraveled significantly altered key metabolic pathways in the diatom following N deprivation. As summarized in Figure 6, it induced genes involved in carbon fixation, the TCA cycle and glycerolipid metabolism, but inhibited genes involved in light harvesting and photosynthesis. Energy resources necessary for carbon fixation and energy demand associated with the neutral lipid synthesis could be replenished from other sources such as elevated TCA cycle. A possible catabolism of carbohydrate reserves including chrysolaminarin may largely explain the neo-synthesis of TAG, in accordance with the up-regulation of glycolysis, coupled with the suppression of critical enzymes, mostly involved in lipid catabolism. The gene expression analyses, in combination with the ultrastructural and biochemical investigations, indicate that additional fatty acids could be produced by membrane remodeling. Thus, the findings provide indications of several mechanisms that contribute to the high lipid contents of microalgal cells under N deprivation.

The results also provide indications of strategies that could be used to enhance biodiesel production from microalgal cultures. The two-step culture method applied here dramatically increased neutral lipid accumulation, up to 2.4-fold, more than previous N limitation or continuous deficiency strategies. Furthermore, the inhibition of light harvesting under N deprivation indicates that high light intensity is not required for lipid accumulation, and the increase in carbon fixation suggests that increasing CO_2_ supplies during this stage should be advantageous.

In summary, results of this study show that diatom cultures with high levels of neutral lipids and increased cell proliferation rates can be obtained by nitrogen deprivation during the late exponential phase. They also provide indications of strategies that could be applied to manipulate the biosynthetic pathways of microalgae to generate cultures with high levels of lipids that may be suitable for biodiesel production.

## Materials and methods

### Algal cultures

Marine diatom *Phaeodactylum tricornutum*, with a fusiform morphotype, was obtained from the Freshwater Algal Culture Collection of the Institute of Hydrobiology, China (No. FACHB-863), and sub-cultured weekly, using a quarter of the preceding cultures as inocula, in Erlenmeyer flasks containing medium sterilized by passage through 0.22-μm filters (Millipore). The growth medium (f/2-Si) contained 75 mg NaNO_3_, 5.65 mg NaH_2_PO_4_ · 2H_2_O, 4.16 mg Na_2_ EDTA, 3.15 mg FeCl_3_ · 6H_2_O, 0.01 mg CuSO_4_ · 5H_2_O, 0.022 mg ZnSO_4_ · 7H_2_O, 0.01 mg CoCl_2_ · 6H_2_O, 0.18 mg MnCl_2_ · 4H_2_O, 0.006 mg Na_2_MoO_4_ · 2H_2_O, 0.0005 mg vitamin B_12_, 0.1 mg vitamin B_1_ and 0.0005 mg Biotin per liter in natural seawater, acquired from Daya Bay, Huizhou, China. The synchronized cultures were routinely cultivated in an artificial climate incubator at constant irradiance (200 μmol photons · m^–2^ · s^–1^) and temperature (21 ± 0.5°C) with 12 h/12 h (1ight/dark) photoperiods.

For the experiments reported here, 1.5 L of algal culture in late-logarithmic growth phase was harvested 5 days after subculturing at 14:00 by centrifugation (4400 rpm for 10 min at 4°C). The supernatant was discarded, the cell pellet was washed twice with NaNO_3_-free f/2-Si medium to completely remove the N. Then the cells were collected by centrifugation (4400 rpm for 15 min at 4°C), and the resulting pellet was re-inoculated into 1.5 L N-free f/2-Si medium. The resuspended culture was divided into two aliquots. One aliquot was split into six equal portions, which were transferred into six flasks, each containing 125 mL N-free medium, and cultured under the above conditions (designated N-deprived cultures). The other aliquot was also divided, transferred to six flasks and similarly treated, except that the medium was supplemented with N, to provide controls. To maintain sampling consistency with respect to the diel cycle, cells were sampled at 14:00 every day.

### Cell density determination

Cell numbers were counted using an Olympus microscope and Brightline Hemocytometer at the same time every day, in triplicates. Cell density (cell mL^-1^) was calculated as follows:

$$\mathrm{CD}=\left(N/80\right)\times 400\times 10^{4}$$

where CD is cell density and N is the cell number per 80 grid cells.

To facilitate counting, each sample was diluted 2-fold if the cell density exceeded 4 × 10^6^ cells mL^-1^.

### Neutral lipid content analysis

Nile red is a soluble phenoxazone lipid dye that partitions to cytoplasmic oil bodies in cells, and becomes fluorescent [41], the intensity of the fluorescence providing robust and convenient indications of the amount of neutral lipids present in various species, including the green microalga *Chlorella*[14,41,42]. In this study, the validity of the method for determining cellular lipid contents of *P. tricornutum* was first tested, by staining samples of the cultures with Nile Red (Sigma) following Chen *et al*., with modifications [14]. Nile red (30 μL of a 0.1 mg mL^-1^ acetone solution) was added to 3 mL portions of cell cultures in triplicate, the resulting suspensions were mixed by rapid inversion, and incubated in darkness for 20 min at room temperature. The stained cell cultures were then transferred to cuvettes to determine their fluorescence intensity using a fluorescence spectrophotometer with 480 nm excitation wavelength. The optimal emission wavelength (592 nm) and staining time (20 min) were determined empirically, using non-stained cultures as auto-fluorescence controls. The relative fluorescence intensity values reported here reflect the differences in neutral lipid contents between stained and non-stained cells.

### Fatty acid composition analysis

For fatty acid profiling analysis, total lipids were extracted from three independent biological replicates according to the method of Lepage and Roy [43], with modifications. Portions (250 mL) of the cultures were harvested after two days of N deprivation by centrifugation at 4400 rpm at 4°C for 10 min. Pellets were transferred to 10 mL tubes. Then 5 mL KOH-CH_3_OH solution was added to each tube, the cells were lysed by ultrasonication in an ice bath, the tubes were infused with nitrogen for 1 min, tightly sealed, agitated and incubated at 75°C for 10 min. After cooling to room temperature the upper phase was transferred to a 50 mL tube. The lower phase was subjected to two further washes with 5 mL KOH-CH_3_OH, lysis and incubation, as described above, the resulting upper phases from each sample were pooled in a single tube, then 15 mL of HCl-CH_3_OH solution was added, the solution was thoroughly mixed and incubated again at 75°C for 10 min. N-hexane (4 mL) was added, the mixture was vortex-mixed, and allowed to settle. The upper phase was then transferred to a new 10 mL tube, while the lower phase was re-extracted with hexane. Then, the upper phases were pooled in a 10 mL tube, dried under a nitrogen stream in a Nitrogen Evaporator (Organomation, USA) and the residues were weighed and stored at −80°C until analysis. Fatty acids were determined by gas chromatography–mass spectrometry (GC-MS) at the Institute of Microbiology, Guangdong, China. The chromatographic column used was a 30 m × 0.25 mm × 0.25 μm DB-5 quartz capillary column. The column temperature was held at 60°C for 1 min, raised by 10°C min^-1^ to 160°C, and to a final temperature of 250°C at 2.5°C min^-1^. The injector temperature was 280°C, and 1 μL samples were injected, splitless. The mass spectrum transmission line temperature was 200°C, and fatty acids were identified using the equipped NBS spectrum library, and quantitatively analyzed by determining integrated peak areas. Relative (percentage) contents of detected fatty acids were calculated by using the normalization method.

For lipid profiling analysis, total algal lipids were extracted following a protocol for *Arabidopsis* leaf tissue [44] with modifications. 200 ml algal culture was harvested and the resulting pellets were quickly immersed in 3 ml preheated (75°C) isopropanol with 0.01% butylated hydroxytoluene (BHT) for 15 min. The algal cells were then subjected to 80 rounds (5 s) of ultrasonication in an Ultrasonic Crasher Noise Isolating Chamber, with 200 W output energy and 7 s intervals, during which the samples were placed in an ice bath to prevent lipid degradation. Then, 1.5 ml chloroform and 0.6 ml water were added to the cell detritus, and the suspensions were vortex-mixed and agitated in a shaking incubator at low temperature at 4°C for 40 min. The mixtures were centrifuged at 4400 rpm for 8 min to separate phases, and the lower phase was transferred to a new 10 ml centrifuge tube. The remaining debris was extracted twice with 3 ml chloroform/methanol (2:1) with 0.01% BHT, after which it was white. All extracts from samples representing cultures subjected to the same treatment were pooled, 1 ml of 1 M KCl was added, the resulting mixture was shaken and centrifuged, and the upper phase was discarded. Then 2 ml water was added to wash the extract, the mixture was centrifuged (12000 rpm for 10 min) and the upper phase was removed. Finally, the organic solvents were evaporated by nitrogen and the residues were stored in a −80°C refrigerator until analysis. Total lipid extracts were separated into lipid classes according to Demandre *et al*. with modifications [45]. The dried algal lipid extracts were re-dissolved in 1 mL buffer A (isopropanol-hexane, 4:3), and the solutions were centrifuged at 14000 rpm for 10 min to remove solid impurities, then transferred to a glass vial. Lipid classes were subsequently separated by HPLC using a silica column and a mobile phase consisting of a 20-min linear gradient from 100% solution A to 100% B (isopropanol-hexane-water, 8:6:1.5), followed by isocratic solution B for a further 25 min, with the column and sample collection system set at 20°C. The total running time, including a 5 min re-equilibration step, was about 50 min. The flow rate was 1.0 mL min^-1^ and 10 μL samples were injected. Eluting fractions of lipids were detected spectrophotometrically at 205 nm, manually collected in separate glass tubes, then stored at −20°C. Compounds in the lipid classes fractionated were diluted in isopropanol and identified using a Micromass Q-TOF micro system (Waters Co., UK) equipped with a syringe pump, LockSpray and ESI interface, operated in positive ionization mode. The mass spectrometer conditions were as follows: electrospray capillary voltage, 3.2 kV; ion source temperature, 110°C; desolvation temperature, 350°C; cone voltage, 15V; cone and collision gases, nitrogen and argon, at 60 L h^-1^ and 600 L h^-1^ flow rates, respectively. Sodium formate solution was used to calibrate the TOF mass spectrometer in positive electrospray ionization (ESI+) mode, and leucine enkephalin (L-EK) was used as an external mass calibration standard. Masses were scanned from 100 to 1100 amu at approximately 0.4 scan s^-1^, and data were collected from m/z 90 to 1000 in continuum mode, and analyzed by Micromass MassLynx 4.1 software (Waters Co., USA). Peaks were also qualitatively analyzed by comparing determined molecular weights with related data in the LIPID MAPS Structure Database (LMSD).

### Observation of oil bodies

To visualize oil bodies and assess their morphology, localization and numbers in the cultured cells Nile Red staining was applied (0.1 mg mL^-1^ in acetone in a 1:100 ratio) in dark conditions, using a similar procedure to that described for determination of the cells’ neutral lipid contents. The mixtures were thoroughly mixed, applied to a glass slide, covered with a coverslip after about 10 min, then observed under an LSM 510 META laser-scanning confocal microscope (Zeiss), with 543 nm excitation wavelength and 570–610 nm emission wavelength. Pictures were acquired randomly from at least 20 cells per sample, and typical images are presented here.

### RNA-Seq analysis

After 48 hours of N deprivation, total RNA was extracted from six replicates of control and N-deprived cultures using an RNeasy Plant Mini kit (QIAgen) following the manufacturer’s instructions. The RNA samples were treated with QIAgen RNase-free DNase I during extraction, and the extracted mRNA was enriched using oligo(dT) magnetic beads. After adding fragmentation buffer, the mRNA was fragmented into short sequences (about 200 bp), first-strand cDNA was synthesized using random hexamer-primers and the mRNA fragments as templates, and then second strands were synthesized. The resulting double-stranded cDNA was purified with a QIAquick PCR extraction kit and subjected to end repair and single nucleotide A (adenine) addition. Finally, sequencing adaptors were ligated to the fragments, the required fragments were purified by agarose gel electrophoresis, enriched by PCR amplification, and sequenced using a HiSeq™ 2000 (Illumina) instrument, with default quality parameters, at the BGI (Shenzheng), China.

### Validation of RNA-Seq data by quantitative real-time PCR

Triplicate portions (2 μg) of total RNA were reverse-transcribed using random hexamer primers and an Omniscript reverse transcription kit (QIAgen). Target genes (and β-actin as a housekeeping marker) were then subjected to quantitative PCR amplification in 96-well optical reaction plates in 20 μL mixtures per well, using a SYBR Green Kit (Takara Bio) following the manufacturer’s instructions and a 7300 Sequence Detection System (Applied Biosystems/Life Technologies). The threshold cycle (Ct) for each well was measured, and the mRNA levels of the target genes in N-deprived cells, relative to those of controls, were quantified after normalization to β-actin.

### RNA-Seq data processing and metabolic pathway analysis

The draft *P. tricornutum* reference genome was obtained from http://genome.jgi-psf.org/Phatr2/Phatr2.download.ftp.html. The sequence is composed of “finished chromosomes” (Phatr2) and “unmapped sequences” (Phatr2_bd), which were annotated separately, and both were included in this study for comprehensive analysis. We aligned RNA short reads (11751810 in total) with the reference genome using SOAP 2.21 and default parameters. All original and mapped data are available at http://guanlab.ccmb.med.umich.edu/data/Yang_N_deprivation/. This resulted in 10786429 mapped reads (91.79%). Any gene with one or no reads was discarded as non-confident, and with this constraint we aligned 12312844 reads obtained from samples of N-deprived cultures, with 11326275 (91.99%) mapped to the genome. RPKM (Reads Per Kilobase per Million mapped reads) values were estimated and log_2_-transformed [46] for each gene defined in the Phatr2 database. This allowed us to estimate the fold-change in their transcript levels under nitrogen deprivation. We downloaded the KEGG pathway database (a unique set of genes that can be confidently associated with each protein in the KEGG pathway repository) released on Jun 30, 2011. We compiled all KEGG-annotated genes for *P. tricornutum*, and all those of other species meeting an e-value cutoff constraint of 1e-5 identified by BLAST analysis, retaining the best hits. We then hand-curated these two sets to remove inconsistencies and incorrect annotations, which resulted in 3648 genes with a KEGG id in total, and transformed the expression fold-change to a color gradient, where red represents up-regulation and green down-regulation. The subcellular localization of the enzymes were based on the prediction in the GenBank database, the prediction softwares online including WoLF PSORT (http://wolfpsort.org/) and SignalP (http://www.cbs.dtu.dk/services/SignalP/), besides relevant references.

### Measurement of photosynthesis activity

Chlorophyll fluorescence parameters sensitively reflect the instantaneous photosynthetic state of diatoms and their acclimation to current environmental conditions. Fv/Fm (the variable/maximum fluorescence ratio) indicates the maximum photochemical quantum yield of PSII reaction centers, reflecting the photosynthetic light energy conversion efficiency. Thus, it is a widely used index of photosynthetic performance and acclimation status [21]. Fo is the minimum fluorescence yield when PSII reaction centers are fully open. Damage to or irreversible loss of activity of PSII reaction centers will cause a decrease in the Fo value. Fm is the maximum fluorescence yield when PSII reaction centers are completely closed, thus it reflects the PSII electron transport capacity. Fv is the variable fluorescence (Fv = Fm-Fo), reflecting reduction of the PSII primary electron acceptor QA, thus indicating the photochemical activity of PSII reaction centers. To measure these parameters, *P. tricornutum* cultures were kept in the dark for 20 min, then exposed to a saturating light pulse (3000 mol · m^-2^ · s^-1^) for l sec while the chlorophyll fluorescence intensities were measured with a Handy-PEA chlorophyll fluorimeter (Hansatech Instruments Ltd) following the manufacturer’s recommendations.

### Ultrastructural analysis by transmission electron microscopy

Samples were fixed in 2% v/v glutaraldehyde, 2% w/v paraformaldehyde in 100 mM sodium cacodylate buffer-NaOH, pH 7.4, for 2.5 h at 4°C and rinsed three times (20 min per rinse) with 130 mM sucrose, 10 mM 2-mercaptoethanol in 100 mM sodium cacodylate buffer-NaOH, pH 7.4. They were then post-fixed with 1% (w/v) osmium tetroxide in 100 mM sodium cacodylate buffer-NaOH, pH 7.4, rinsed three times (5 min per rinse) with ultrapure (Milli-Q) water and dehydrated through a graded series of acetone (20%, 50%, 70%, 90%, 100% v/v). After infiltration through a graded acetone/Epon/Spurr’s epoxy resin series, samples were embedded in 100% w/v Spurr’s epoxy resin and polymerized at 60°C for 24 h. Ultrathin sections were prepared using a Diatome diamond knife on an 8800 Ultratome III (LKB Instruments) and stained with uranyl acetate and lead citrate. The stained sections (about 20 per treatment) were examined under a JEM-1200EX transmission electron microscope (JEOL), and images were recorded on 4489 film (Eastman-Kodak).

## Abbreviations

RNA-seq: RNA-sequencing; MGDG: Monogalactosyldiacylglycerol; TCA: Tricarboxylic cycle; TAG: Triacylglycerol; PUFA: Polyunsaturated fatty acid; SFA: Saturated fatty acid; KEGG: Kyoto encyclopedia of genes and genomes; LHC: Light harvesting complex; PSII: Photosystem II; ROS: Reactive oxygen species; PEPC: Phosphoenolpyruvate carboxylase; OAA: Oxaloacetate; CCM: Carbon concentrating mechanism; GS: Glutamine synthetase; PEP: Phosphoenolpyruvate; ME: Malic enzyme; ACCase: Adetyl-CoA carboxylase; FAS: Fatty acid synthase; FTIR: Fourier transform infrared spectrometer; FBP: Fructose-1,6-bisphosphatase; DGAT: Diacylglycerol acyltransferase; PDAT: Phospholipid:diacylglycerol acyltransferase; PC: Phosphatidylcholine; RPKM: Reads per kilobase per million mapped reads; FA: Fatty acid; DW: Dry weight.

## Competing interests

The authors declare that there are no competing interests.

## Authors’ contributions

HL and YG designed the research; ZY, YN, YM, JX, MZ, WY, JL, YG and HL performed research; YG, ZY and HL contributed analytic and computational tools; ZY, YN, WY, JL, SL, YG and HL analyzed data; ZY, YG and HL wrote the paper. All authors read and approved the final manuscript.

## Supplementary Material

Additional file 1 **DataSet of gene expression of *P. tricornutum *following N deprivation.** Click here for file

Additional file 2: Figure S1 Distribution of the number of reads across different mRNA sequence size. Each box plot depicts the numbers of reads for each gene (log base 10) in an mRNA sequence size bin (0–5,000 bp, bin size of 500 bp). The line shows the range from minimum to maximum. The box and bar shows the quantiles. All mRNAs of 5,000 or more nucleotides are classified as 5,000 and above. A) nitrogen deprived condition. B) normal condition. Click here for file

Additional file 3: Table S1 Fold changes in the expression of some genes encoding enzymes involved in various metabolisms following N deprivation. Click here for file

Additional file 4: Figure S2 Expression alteration in some metabolic pathways. A) nitrogen fixation pathway; B) carbon fixation pathway; C) TCA cycle. Genes were mapped to KEGG pathways through annotation records in KEGG as well as homology map. Numbers in the boxes represent the EC number of the gene. Red indicates increase in expression level and blue indicates decrease. The intensity of the color bar is linearly correlated to the expression level change (log_2_ fold). Click here for file

Additional file 5: Figure S3 Separation and identification of lipid classes. Chromatograms showing (A) Total lipids of control cells, (B) Total lipids of N-deprived cells. Peak identifications are described in the text. Click here for file
