# Supplementary figures and images for: Molecular and cellular mechanisms of neutral lipid accumulation in diatom following nitrogen deprivation
Source: Biotechnol Biofuels. 2013 May 4;6:67. doi: 10.1186/1754-6834-6-67 (PMC3662598; doi:10.1186/1754-6834-6-67)

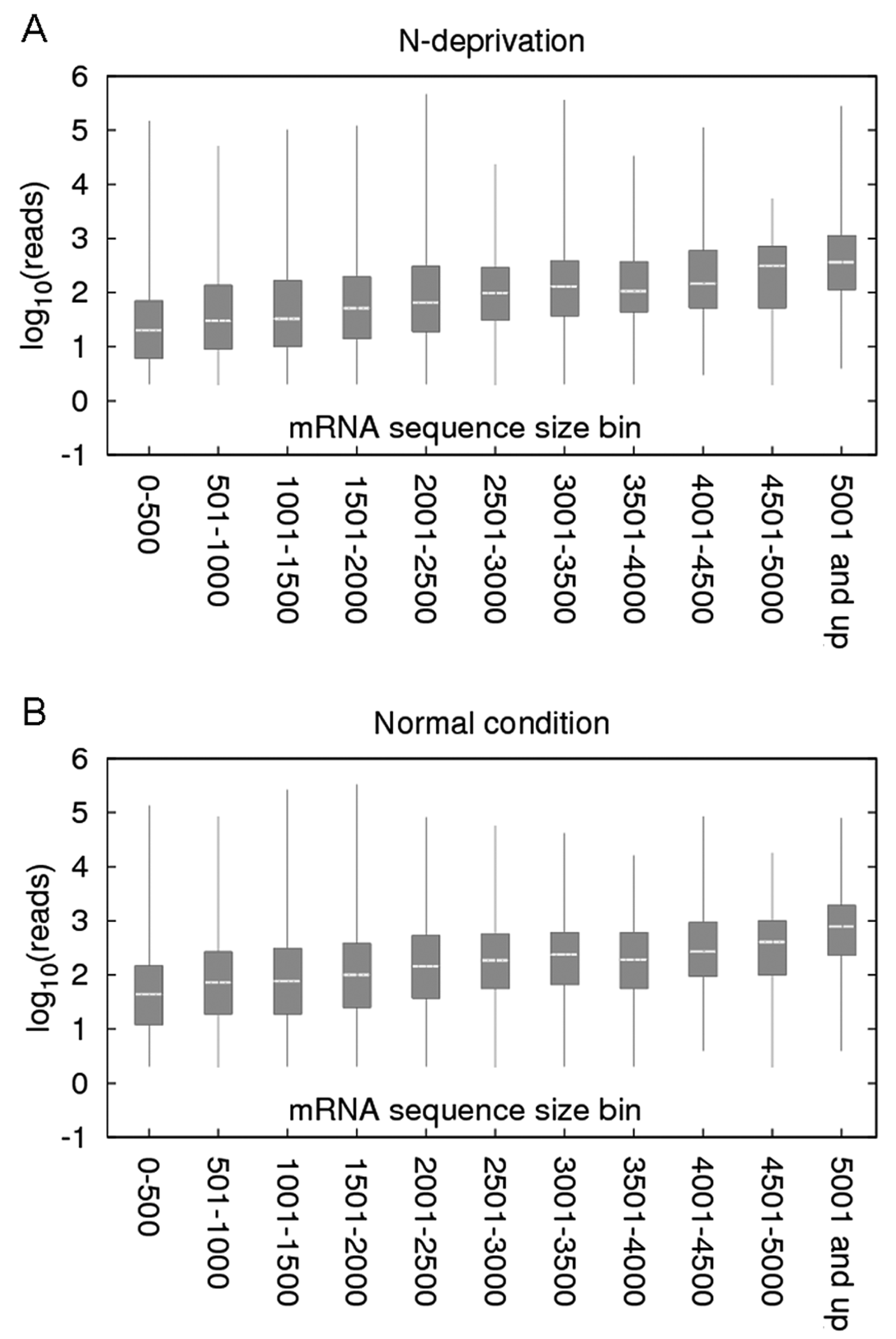

Supplement: Additional file 2: Figure S1 — Distribution of the number of reads across different mRNA sequence size. Each box plot depicts the numbers of reads for each gene (log base 10) in an mRNA sequence size bin (0–5,000 bp, bin size of 500 bp). The line shows the range from minimum to maximum. The box and bar shows the quantiles. All mRNAs of 5,000 or more nucleotides are classified as 5,000 and above. A) nitrogen deprived condition. B) normal condition. [file 1754-6834-6-67-S2.tiff]

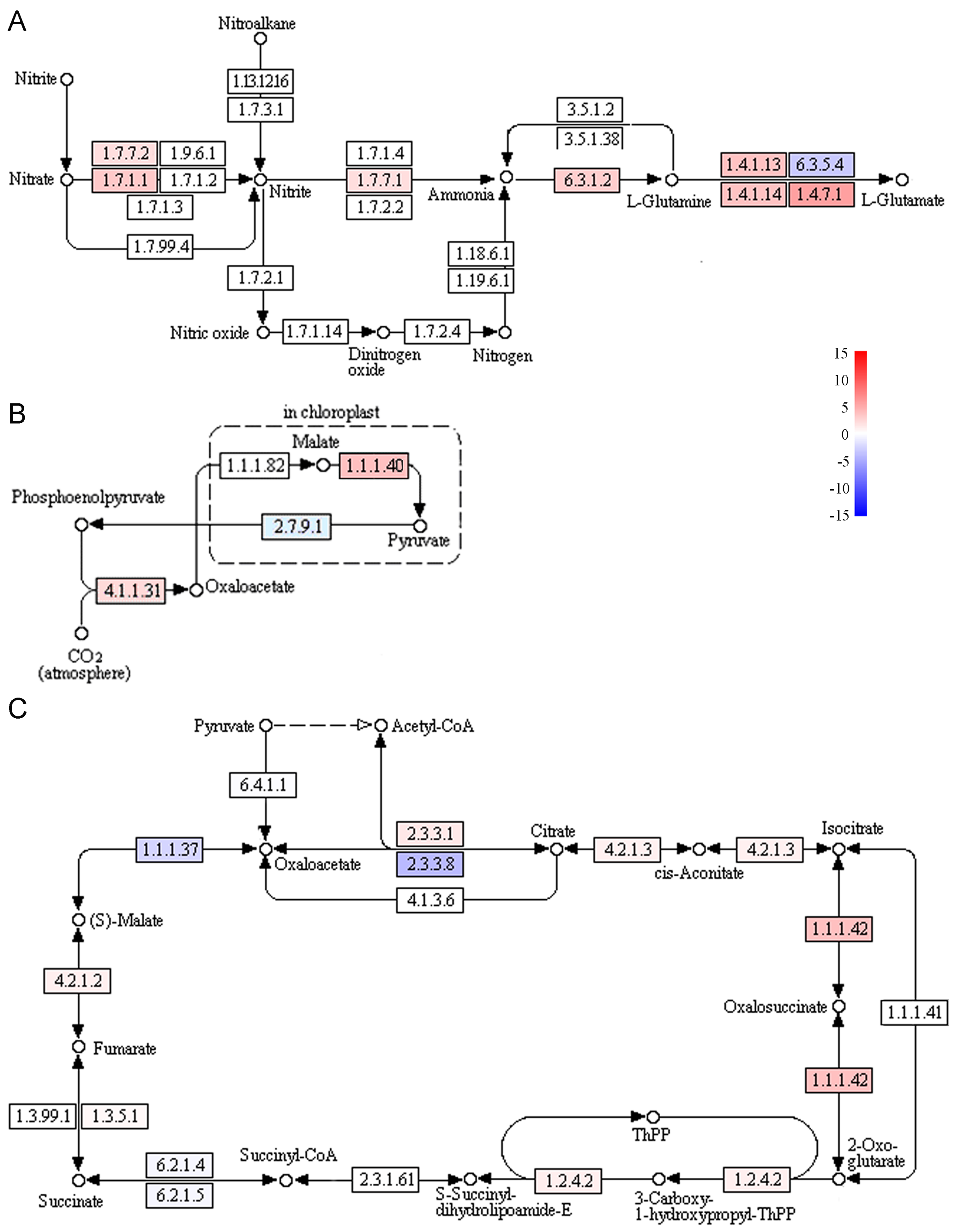

Supplement: Additional file 4: Figure S2 — Expression alteration in some metabolic pathways. A) nitrogen fixation pathway; B) carbon fixation pathway; C) TCA cycle. Genes were mapped to KEGG pathways through annotation records in KEGG as well as homology map. Numbers in the boxes represent the EC number of the gene. Red indicates increase in expression level and blue indicates decrease. The intensity of the color bar is linearly correlated to the expression level change (log2 fold). [file 1754-6834-6-67-S4.tiff]

## Slide 1
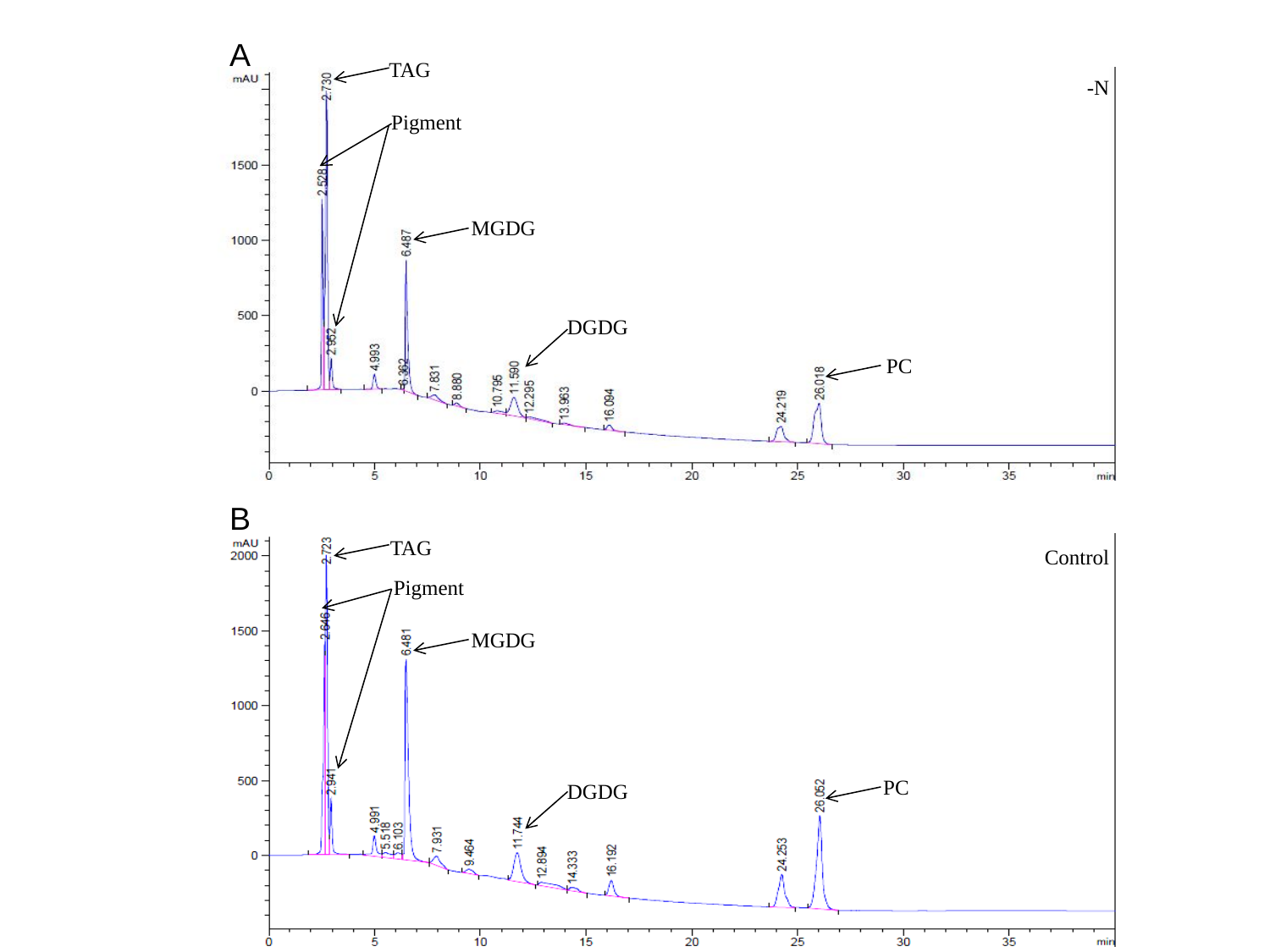

A
B
TAG
-N
Pigment
MGDG
DGDG
PC
TAG
Control
Pigment
MGDG
PC
DGDG

Supplement: Additional file 5: Figure S3 — Separation and identification of lipid classes. Chromatograms showing (A) Total lipids of control cells, (B) Total lipids of N-deprived cells. Peak identifications are described in the text. [file 1754-6834-6-67-S5.ppt]
