# Supplementary material for: Molecular and cellular mechanisms of neutral lipid accumulation in diatom following nitrogen deprivation
Source: Biotechnol Biofuels. 2013 May 4;6:67. doi: 10.1186/1754-6834-6-67 (PMC3662598; doi:10.1186/1754-6834-6-67)
Supplement: Additional file 3: Table S1 — Fold changes in the expression of some genes encoding enzymes involved in various metabolisms following N deprivation. [file 1754-6834-6-67-S3.doc]

**Table S. Fold Changes in the Expression of some Genes Encoding Enzymes Involved in Varoius Metabolisms Following N Deprivation.**

|  | **Locus tag** | | | | | **Annotation** | | | **Ctr RPKM** | | **-N RPKM** | | | **Log2(N/Ctr)** | | | | | | | | **FDR** | | | |  | | |
| --- | --- | --- | --- | --- | --- | --- | --- | --- | --- | --- | --- | --- | --- | --- | --- | --- | --- | --- | --- | --- | --- | --- | --- | --- | --- | --- | --- | --- |
|  | **a) Primary metabolism** | | | | | | | | | | | | | | | | | | | | | | | | |  | | |
|  | 54926 | | | | | acetyl-CoA carboxylase | | | 37.02 | | 6.57 | | | -2.49 | | | | | | | | 3.34e-275 | | | |  | | |
|  | 23871 | | | | | aspartate transaminase | | | 19.77 | | 9.79 | | | -1.01 | | | | | | | | 1.59e-09 | | | |  | | |
|  | 23059 | | | | | aspartate transaminase | | | 1.15 | | 1.19 | | | 0.04 | | | | | | | | 9.60e-01 | | | |  | | |
|  | 30145 | | | | | ATP-citrate synthase | | | 41.56 | | 113.62 | | | 1.45 | | | | | | | | 8.67e-13 | | | |  | | |
|  | 54477 | | | | | ATP-citrate synthase | | | 46.50 | | 2.33 | | | -4.32 | | | | | | | | 3.83e-298 | | | |  | | |
|  | 8860 | | | | | diacylglycerol acyltransferase | | | 323.97 | | 613.93 | | | 0.92 | | | | | | | | 2.06e-11 | | | |  | | |
|  | 43018 | | | | | ferredoxin | | | 26.43 | | 5.71 | | | -2.21 | | | | | | | | 1.79e-86 | | | |  | | |
|  | 2793 | | | | | fructose-1,6-bisphosphatase | | | 54.02 | | 1.21 | | | -5.47 | | | | | | | | 2.16e-126 | | | |  | | |
|  | 9359 | | | | | fructose-1,6-bisphosphatase | | | 24.55 | | 1.44 | | | -4.09 | | | | | | | | 9.40e-49 | | | |  | | |
|  | 31994 | | | | | fructose-1,6-bisphosphatase | | | 5.51 | | 1.86 | | | -1.57 | | | | | | | | 4.54e-05 | | | |  | | |
|  | 23247 | | | | | fructose-1,6-bisphosphatase | | | 5.15 | | 7.79 | | | 0.60 | | | | | | | | 3.31e-02 | | | |  | | |
|  | 8744 | | | | | fructose-1,6-bisphosphatase/plastidic inositol phosphatase | | | 3.47 | | 0.99 | | | -1.80 | | | | | | | | 6.44e-04 | | | |  | | |
|  | 15495 | | | | | glucokinase | | | 32.57 | | 14.56 | | | -1.16 | | | | | | | | 2.54e-10 | | | |  | | |
|  | 52138 | | | | | glucose-6-phosphate dehydrogenase | | | 33.57 | | 46.17 | | | 0.46 | | | | | | | | 1.41e-06 | | | |  | | |
|  | 30040 | | | | | glucose-6-phosphate dehydrogenase | | | 20.09 | | 3.05 | | | -2.72 | | | | | | | | 4.73e-50 | | | |  | | |
|  | 45239 | | | | | glutamate dehydrogenase | | | 558.41 | | 41.54 | | | -3.75 | | | | | | | | 0.00e+00 | | | |  | | |
|  | 3262 | | | | | glycerol 3-phosphate acyltransferase | | | 1.85 | | 12.02 | | | 2.70 | | | | | | | | 2.47e-06 | | | |  | | |
|  | 8975 | | | | | glycerol-3-phosphate dehydrogenase | | | 1.86 | | 0.11 | | | -4.07 | | | | | | | | 1.69e-04 | | | |  | | |
|  | 14401 | | | | | isocitrase | | | 105.56 | | 21.67 | | | -2.28 | | | | | | | | 1.56e-179 | | | |  | | |
|  | 54738 | | | | | isomerase triosephosphate isomerase | | | 4143.97 | | 709.97 | | | -2.55 | | | | | | | | 0.00e+00 | | | |  | | |
|  | 54998 | | | | | kinase pyruvate kinase 3 | | | 47.87 | | 1.22 | | | -5.29 | | | | | | | | 2.27e-179 | | | |  | | |
|  | 40261 | | | | | phosphatidate phosphatase | | | 26.49 | | 33.02 | | | 0.32 | | | | | | | | 2.50e-02 | | | |  | | |
|  | 39949 | | | | | phosphatidate phosphatase | | | 5.14 | | 1.68 | | | -1.61 | | | | | | | | 3.59e-04 | | | |  | | |
|  | 16844 | | | | | phosphofructokinase | | | 182.48 | | 269.38 | | | 0.56 | | | | | | | | 1.35e-11 | | | |  | | |
|  | 55126 | | | | | pyrophosphate-dependent phosphofructose kinase | | | 1650.57 | | 2256.29 | | | 0.45 | | | | | | | | 0.00e+00 | | | |  | | |
|  | 14284 | | | | | pyrophosphate-dependent phosphofructose kinase | | | 52.82 | | 19.20 | | | -1.46 | | | | | | | | 1.46e-37 | | | |  | | |
|  | 22404 | | | | | pyruvate kinase | | | 2.83 | | 0.24 | | | -3.56 | | | | | | | | 3.90e-10 | | | |  | | |
|  | 55079 | | | | | pyruvate kinase | | | 0.97 | | 6.24 | | | 2.68 | | | | | | | | 1.11e-07 | | | |  | | |
|  | 49098 | | | | | pyruvate kinase 2 | | | 0.82 | | 0.52 | | | -0.65 | | | | | | | | 3.75e-01 | | | |  | | |
|  | 45997 | | | | | pyruvate kinase 4a | | | 0.14 | | 3.60 | | | 4.71 | | | | | | | | 1.87e-04 | | | |  | | |
|  | 46001 | | | | | pyruvate kinase 4b | | | 0.24 | | 2.09 | | | 3.10 | | | | | | | | 7.35e-05 | | | |  | | |
|  | 22913 | | | | | pyruvate kinase 5 | | | 2.49 | | 1.36 | | | -0.87 | | | | | | | | 3.91e-02 | | | |  | | |
|  | 13382 | | | | | ribose 5-phosphate isomerase | | | 91.74 | | 18.41 | | | -2.32 | | | | | | | | 1.09e-72 | | | |  | | |
|  | 18228 | | | | | triosephosphate isomerase | | | 11.54 | | 6.00 | | | -0.94 | | | | | | | | 2.82e-04 | | | |  | | |
|  | 50738 | | | | | triosephosphate isomerase | | | 0.51 | | 0.24 | | | -1.07 | | | | | | | | 4.66e-01 | | | |  | | |
|  | **b) Nitrogen metabolism** | | | | | | | | | | | | | | | | | | | | | | | | |  | | |
| 1862 | | | | | | ammonium transporter | | 37.79 | | | 69.00 | | | 0.87 | | | | | 0 | | | | | | |  | | |
| 10881 | | | | | | ammonium transporter | | 32.37 | | | 5.64 | | | 0.87 | | | | | 1.35e-04 | | | | | | |  | | |
| 11128 | | | | | | ammonium transporter | | 0.18 | | | / | | | / | | | | | 2.75e-01 | | | | | | |  | | |
| 13418 | | | | | | ammonium transporter | | 2.05 | | | 7.47 | | | 1.87 | | | | | 7.19e-09 | | | | | | |  | | |
| 27877 | | | | | | ammonium transporter | | 32.81 | | | 1440.66 | | | 5.46 | | | | | 0 | | | | | | |  | | |
| 45443 | | | | | | carbonic anhydrase | | 13.07 | | | 6.97 | | | -0.91 | | | | | 9.19e-05 | | | | | | |  | | |
| 15917 | | | | | | cystathionine gamma-lyase | | 30.36 | | | 4.35 | | | -2.80 | | | | | 2.60e-48 | | | | | | |  | | |
| 51214 | | | | | | ferredoxin-dependent glutamate synthase | | 8.88 | | | 128.98 | | | 3.86 | | | | | 1.31e-12 | | | | | | |  | | |
| 12902 | | | | | | ferredoxin--nitrite reductase | | 3.94 | | | 16.80 | | | 2.09 | | | | | 1.31e-13 | | | | | | |  | | |
| 37719 | | | | | | formamidase-like protein | | 0.31 | | | 4.98 | | | 3.99 | | | | | 1.18e-04 | | | | | | |  | | |
| 54476 | | | | | | formidase | | 1.34 | | | 2.74 | | | 1.04 | | | | | 2.39e-03 | | | | | | |  | | |
| 22357 | | | | | | GLNA, glutamine synthase | | 22.70 | | | 150.78 | | | 2.73 | | | | | 4.30e-12 | | | | | | |  | | |
| 50971 | | | | | | glutamate dehydrogenase | | 119.35 | | | 26.42 | | | -2.18 | | | | | 1.03e-241 | | | | | | |  | | |
| 46384 | | | | | | glycine decarboxylase t-protein | | 41.49 | | | 9.10 | | | -2.19 | | | | | 4.46e-111 | | | | | | |  | | |
| 54983 | | | | | | nitrate reductase | | 1.59 | | | 53.46 | | | 5.08 | | | | | 3.69e-13 | | | | | | |  | | |
| 45239 | | | | | | predicted protein | | 558.41 | | | 41.54 | | | -3.75 | | | | | 0.00e+00 | | | | | | |  | | |
| 13951 | | | | | | predicted protein | | 1092.85 | | | 2189.32 | | | 1.00 | | | | | 0.00e+00 | | | | | | |  | | |
| 24739 | | | | | | predicted protein | | 0.42 | | | 26.34 | | | 5.98 | | | | | 3.11e-08 | | | | | | |  | | |
| 35395 | | | | | | predicted protein | | 244.30 | | | 1413.55 | | | 2.53 | | | | | 1.12e-11 | | | | | | |  | | |
| 30770 | | | | | | predicted protein | | 4.11 | | | 26.21 | | | 2.67 | | | | | 8.56e-09 | | | | | | |  | | |
| 20905 | | | | | | predicted protein | | 6.10 | | | 0.66 | | | -3.22 | | | | | 7.66e-16 | | | | | | |  | | |
| 55029 | | | | | | predicted protein | | 27.57 | | | 2.38 | | | -3.53 | | | | | 1.11e-56 | | | | | | |  | | |
| 35370 | | | | | | predicted protein | | 1.92 | | | 0.72 | | | -1.42 | | | | | 1.33e-02 | | | | | | |  | | |
| 17187 | | | | | | predicted protein | | 52.66 | | | 0.95 | | | -5.80 | | | | | 1.32e-88 | | | | | | |  | | |
| 44902 | | | | | | predicted protein | | 374.33 | | | 108.63 | | | -1.78 | | | | | 0.00e+00 | | | | | | |  | | |
| 51092 | | | | | | predicted protein | | 1.07 | | | 13.05 | | | 3.60 | | | | | 1.34e-06 | | | | | | |  | | |
| 18337 | | | | | | predicted protein | | 1.13 | | | 0.11 | | | -3.39 | | | | | 7.36e-05 | | | | | | |  | | |
| 20342 | | | | | | synthase of glutamate synthase | | 62.22 | | | 37.13 | | | -0.74 | | | | | 4.84e-23 | | | | | | |  | | |
| **c) Photosynthesis** | | | | | | | | | | | | | | | | | | | | | | | | |  | | | |
| 44056 | | | cytochrome c6, cytochrome c553 | | | | 238.09 | | | | | | 6.19 | -5.27 | | | | | | | 0.00e+00 | | | | | | |  |
| 42519 | | | fucoxanhin chlorophyll binding protein related | | | | 6.52 | | | | | | 3.63 | -0.84 | | | | | | | 4.23e-02 | | | | | | |  |
| 25893 | | | fucoxanthin chlorophyll a | | | | 341.47 | | | | | | 26.65 | -3.68 | | | | | | | 0.00e+00 | | | | | | |  |
| 17531 | | | fucoxanthin chlorophyll a | | | | 176.74 | | | | | | 2.61 | -6.08 | | | | | | | 2.16e-272 | | | | | | |  |
| 48797 | | | fucoxanthin chlorophyll a | | | | 60.52 | | | | | | 5.74 | -3.40 | | | | | | | 5.06e-170 | | | | | | |  |
| 23257 | | | fucoxanthin chlorophyll a | | | | 153.92 | | | | | | 9.07 | -4.09 | | | | | | | 9.70e-299 | | | | | | |  |
| 48882 | | | fucoxanthin chlorophyll a | | | | 789.33 | | | | | | 3.22 | -7.94 | | | | | | | 0.00e+00 | | | | | | |  |
| 22680 | | | fucoxanthin chlorophyll a | | | | 238.86 | | | | | | 11.75 | -4.34 | | | | | | | 0.00e+00 | | | | | | |  |
| 54027 | | | fucoxanthin chlorophyll a | | | | 154.25 | | | | | | 18.71 | -3.04 | | | | | | | 4.31e-196 | | | | | | |  |
| 16481 | | | fucoxanthin chlorophyll a | | | | 245.42 | | | | | | 2499.53 | 3.35 | | | | | | | 6.73e-12 | | | | | | |  |
| 22956 | | | fucoxanthin chlorophyll a | | | | 77.54 | | | | | | 13.20 | -2.55 | | | | | | | 3.02e-80 | | | | | | |  |
| 9799 | | | fucoxanthin chlorophyll a | | | | 112.47 | | | | | | 8.53 | -3.72 | | | | | | | 1.73e-96 | | | | | | |  |
| 14442 | | | fucoxanthin chlorophyll a | | | | 113.17 | | | | | | 23.15 | -2.29 | | | | | | | 2.09e-49 | | | | | | |  |
| 14386 | | | fucoxanthin chlorophyll a | | | | 84.18 | | | | | | 0.95 | -6.46 | | | | | | | 4.69e-97 | | | | | | |  |
| 38720 | | | fucoxanthin chlorophyll a | | | | 46.07 | | | | | | 58.22 | 0.34 | | | | | | | 8.71e-03 | | | | | | |  |
| 27278 | | | fucoxanthin chlorophyll a | | | | 28.90 | | | | | | 42.50 | 0.56 | | | | | | | 3.56e-05 | | | | | | |  |
| 25168 | | | fucoxanthin chlorophyll a | | | | 18.95 | | | | | | 0.88 | -4.42 | | | | | | | 2.63e-30 | | | | | | |  |
| 54065 | | | fucoxanthin chlorophyll a | | | | 9.58 | | | | | | 1.12 | -3.09 | | | | | | | 3.17e-11 | | | | | | |  |
| 50705 | | | fucoxanthin chlorophyll a | | | | 4.65 | | | | | | 0.31 | -3.92 | | | | | | | 2.61e-07 | | | | | | |  |
| 44733 | | | fucoxanthin chlorophyll a | | | | 4.05 | | | | | | 1.57 | -1.36 | | | | | | | 9.04e-03 | | | | | | |  |
| 14242 | | | fucoxanthin chlorophyll a | | | | 3.12 | | | | | | 3.37 | 0.11 | | | | | | | 8.51e-01 | | | | | | |  |
| 11006 | | | fucoxanthin chlorophyll a | | | | 401.38 | | | | | | 13.80 | -4.86 | | | | | | | 0.00e+00 | | | | | | |  |
| 17766 | | | fucoxanthin chlorophyll a | | | | 183.89 | | | | | | 11.93 | -3.95 | | | | | | | 4.02e-274 | | | | | | |  |
| 20331 | | | oxygen-evolving enhancer protein 1 precursor | | | | 774.92 | | | | | | 103.18 | -2.91 | | | | | | | 0.00e+00 | | | | | | |  |
| 55057 | | | photosystem II subunit | | | | 1269.35 | | | | | | 99.47 | -3.67 | | | | | | | 0.00e+00 | | | | | | |  |
| 20657 | | | precursor of ATPase ATPase gamma subunit | | | | 8.38 | | | | | | 7.05 | -0.25 | | | | | | | 3.21e-01 | | | | | | |  |
| 31465 | | | predicted protein | | | | 1.26 | | | | | | 2.34 | 0.90 | | | | | | | 2.56e-02 | | | | | | |  |
| 46657 | | | predicted protein | | | | 92.62 | | | | | | 17.01 | -2.44 | | | | | | | 6.95e-81 | | | | | | |  |
| 13358 | | | predicted protein | | | | 52.54 | | | | | | 21.28 | -1.30 | | | | | | | 1.41e-14 | | | | | | |  |
| 23717 | | | predicted protein | | | | 5.62 | | | | | | 1.67 | -1.75 | | | | | | | 2.21e-06 | | | | | | |  |
| 12813 | | | predicted protein | | | | 2.04 | | | | | | 0.26 | -2.97 | | | | | | | 1.48e-03 | | | | | | |  |
| 42018 | | | predicted protein | | | | 1.40 | | | | | | 0.24 | -2.53 | | | | | | | 1.45e-02 | | | | | | |  |
| 15777 | | | predicted protein | | | | 0.95 | | | | | | 5.18 | 2.45 | | | | | | | 1.69e-05 | | | | | | |  |
| 48359 | | | predicted protein | | | | 38.85 | | | | | | 0.29 | -7.07 | | | | | | | 7.17e-76 | | | | | | |  |
| 26293 | | | predicted protein | | | | 232.16 | | | | | | 14.90 | -3.96 | | | | | | | 4.45e-268 | | | | | | |  |
| 9078 | | | predicted protein | | | | 908.06 | | | | | | 11.74 | -6.27 | | | | | | | 0.00e+00 | | | | | | |  |
| 15820 | | | predicted protein | | | | 10.40 | | | | | | 6.24 | -0.74 | | | | | | | 7.68e-02 | | | | | | |  |
| 6062 | | | predicted protein | | | | 467.32 | | | | | | 30.67 | -3.93 | | | | | | | 0.00e+00 | | | | | | |  |
| **d) Carbohydrate metabolism** | | | | | | | | | | | | | | | | | | | | | | | | |  | | | |
| 34010 | | | | alanine aminotransferase | | | 82.55 | | | | | 17.15 | | | | -2.27 | | | | | | 6.46e-123 | | | | | | |
| 29014 | | | | cytosolic class II aldolase | | | 382.57 | | | | | 555.88 | | | | 0.54 | | | | | | 1.88e-11 | | | | | | |
| 54834 | | | | dehydrogenase | | | 15.11 | | | | | 1.02 | | | | -3.89 | | | | | | 4.90e-35 | | | | | | |
| 2793 | | | | fructose-1,6-bisphosphatase | | | 54.02 | | | | | 1.21 | | | | -5.47 | | | | | | 2.16e-126 | | | | | | |
| 9359 | | | | fructose-1,6-bisphosphatase | | | 24.55 | | | | | 1.44 | | | | -4.09 | | | | | | 9.40e-49 | | | | | | |
| 23247 | | | | fructose-1,6-bisphosphatase | | | 5.15 | | | | | 7.79 | | | | 0.60 | | | | | | 3.31e-02 | | | | | | |
| 31994 | | | | fructose-1,6-bisphosphatase | | | 5.51 | | | | | 1.86 | | | | -1.57 | | | | | | 4.54e-05 | | | | | | |
| 22993 | | | | fructose-1,6-bisphosphate aldolase | | | 116.20 | | | | | 3.35 | | | | -5.12 | | | | | | 0.00e+00 | | | | | | |
| 41423 | | | | fructose-bisphosphate aldolase | | | 254.85 | | | | | 268.44 | | | | 0.07 | | | | | | 9.55e-02 | | | | | | |
| 54738 | | | | isomerase triosephosphate isomerase | | | 4143.97 | | | | | 709.97 | | | | -2.55 | | | | | | 0.00e+00 | | | | | | |
| 55079 | | | | kinase pyruvate kinase | | | 0.97 | | | | | 6.24 | | | | 2.68 | | | | | | 1.11e-07 | | | | | | |
| 49098 | | | | kinase pyruvate kinase 2 | | | 0.82 | | | | | 0.52 | | | | -0.65 | | | | | | 3.75e-01 | | | | | | |
| 54998 | | | | kinase pyruvate kinase 3 | | | 47.87 | | | | | 1.22 | | | | -5.29 | | | | | | 2.27e-179 | | | | | | |
| 45997 | | | | kinase pyruvate kinase 4a | | | 0.14 | | | | | 3.60 | | | | 4.71 | | | | | | 1.87e-04 | | | | | | |
| 46001 | | | | kinase pyruvate kinase 4b | | | 0.24 | | | | | 2.09 | | | | 3.10 | | | | | | 7.35e-05 | | | | | | |
| 22913 | | | | kinase pyruvate kinase 5 | | | 2.49 | | | | | 1.36 | | | | -0.87 | | | | | | 3.91e-02 | | | | | | |
| 55018 | | | | phosphoenolpyruvate carboxykinase | | | 2539.90 | | | | | 2032.80 | | | | -0.32 | | | | | | 1.40e-208 | | | | | | |
| 10208 | | | | phosphoribulokinase | | | 4.98 | | | | | 4.43 | | | | -0.17 | | | | | | 6.29e-01 | | | | | | |
| 8744 | | | | plastidic inositol phosphatase | | | 3.47 | | | | | 0.99 | | | | -1.80 | | | | | | 6.44e-04 | | | | | | |
| 12668 | | | | predicted protein | | | 17.78 | | | | | 0.73 | | | | -4.61 | | | | | | 1.15e-23 | | | | | | |
| 14792 | | | | predicted protein | | | 1767.46 | | | | | 398.36 | | | | -2.15 | | | | | | 0.00e+00 | | | | | | |
| 27976 | | | | predicted protein | | | 5.83 | | | | | 8.86 | | | | 0.60 | | | | | | 6.99e-05 | | | | | | |
| 42398 | | | | predicted protein | | | 40.84 | | | | | 6.58 | | | | -2.63 | | | | | | 3.33e-50 | | | | | | |
| 48983 | | | | predicted protein | | | 4.04 | | | | | 0.90 | | | | -2.17 | | | | | | 7.02e-12 | | | | | | |
| 51136 | | | | predicted protein | | | 0.76 | | | | | 1.49 | | | | 0.98 | | | | | | 2.77e-02 | | | | | | |
| 51970 | | | | predicted malic enzyme | | | 937.73 | | | | | 6777.65 | | | | 2.85 | | | | | | 0.00e+00 | | | | | | |
| 54082 | | | | predicted protein | | | 1.45 | | | | | 1.52 | | | | 0.07 | | | | | | 8.97e-01 | | | | | | |
| 22404 | | | | pyruvate kinase | | | 2.83 | | | | | 0.24 | | | | -3.56 | | | | | | 3.90e-10 | | | | | | |
| 21988 | | | | pyruvate phosphate dikinase | | | 2362.85 | | | | | 325.54 | | | | -2.86 | | | | | | 0.00e+00 | | | | | | |
| 13382 | | | | Ribose 5-phosphate epimerase | | | 91.74 | | | | | 18.41 | | | | -2.32 | | | | | | 1.09e-72 | | | | | | |
| 20015 | | | | ribulose-phosphate 3-epimerase | | | 47.84 | | | | | 29.05 | | | | -0.72 | | | | | | 2.67e-08 | | | | | | |
| 9124 | | | | seduheptulose bisphosphatase | | | 7.12 | | | | | 1.91 | | | | -1.90 | | | | | | 7.05e-07 | | | | | | |
| 29260 | | | | transketolase | | | 16.02 | | | | | 5.42 | | | | -1.56 | | | | | | 1.86e-24 | | | | | | |
| 41856 | | | | transketolase | | | 17.94 | | | | | 4.44 | | | | -2.01 | | | | | | 6.19e-33 | | | | | | |
| 18228 | | | | triosephosphate isomerase | | | 11.54 | | | | | 6.00 | | | | -0.94 | | | | | | 2.82e-04 | | | | | | |
| 50738 | | | | triosephosphate isomerase | | | 0.51 | | | | | 0.24 | | | | -1.07 | | | | | | 4.66e-01 | | | | | | |
| **e) TCA cycle** | | | | | | | | | | | | | | | | | | | | | | | | |  | | | |
| 29016 | | 2-oxoglutarate dehydrogenase E1 component | | | | | 3.74 | | | | 4.49 | | | | 0.27 | | | | | 1.79e-01 | | | | | | |  | |
| 54477 | | ATP-citrate synthase | | | | | 46.50 | | | | 2.33 | | | | -4.32 | | | | | 3.83e-298 | | | | | | |  | |
| 26921 | | beta chain succinyl-coa synthetase synthetase | | | | | 49.95 | | | | 74.49 | | | | 0.58 | | | | | 1.47e-12 | | | | | | |  | |
| 13894 | | dihydrolipoamide acetyl transferase | | | | | 6.16 | | | | 7.58 | | | | 0.30 | | | | | 2.48e-01 | | | | | | |  | |
| 23850 | | dihydrolipoamide acetyl transferase | | | | | 0.90 | | | | 0.14 | | | | -2.65 | | | | | 8.31e-03 | | | | | | |  | |
| 17401 | | dihydrolipoamide acetyltransferase | | | | | 13.81 | | | | 22.98 | | | | 0.73 | | | | | 4.99e-08 | | | | | | |  | |
| 40430 | | dihydrolipoamide succinyltransferase | | | | | 67.75 | | | | 87.01 | | | | 0.36 | | | | | 3.51e-06 | | | | | | |  | |
| 26432 | | dihydrolipoyl dehydrogenase | | | | | 79.72 | | | | 49.73 | | | | -0.68 | | | | | 6.86e-22 | | | | | | |  | |
| 36139 | | fumarate hydratase | | | | | 49.69 | | | | 1.85 | | | | -4.75 | | | | | 3.42e-142 | | | | | | |  | |
| 51720 | | fumarate reductase flavoprotein | | | | | 48.75 | | | | 99.89 | | | | 1.03 | | | | | 6.54e-13 | | | | | | |  | |
| 14762 | | isocitrate dehydrogenase | | | | | 1.03 | | | | 16.23 | | | | 3.98 | | | | | 4.51e-06 | | | | | | |  | |
| 42015 | | ligase succinate-coa ligase | | | | | 49.65 | | | | 33.11 | | | | -0.58 | | | | | 7.04e-07 | | | | | | |  | |
| 30113 | | lipoamide dehydrogenase | | | | | 3.18 | | | | 0.19 | | | | -4.10 | | | | | 4.82e-12 | | | | | | |  | |
| 30519 | | precursor of carboxylase pyruvate carboxylase | | | | | 706.02 | | | | 198.09 | | | | -1.83 | | | | | 0.00e+00 | | | | | | |  | |
| 49339 | | precursor of carboxylase pyruvate carboxylase | | | | | 0.85 | | | | 2.19 | | | | 1.37 | | | | | 1.72e-05 | | | | | | |  | |
| 55035 | | precursor of dehydrogenase pyruvate dehydrogenase E1 component alpha subunit | | | | | 6.90 | | | | 7.93 | | | | 0.20 | | | | | 1.94e-01 | | | | | | |  | |
| 20183 | | precursor of dehydrogenase pyruvate dehydrogenase E1 component beta subunit | | | | | 13.84 | | | | 39.37 | | | | 1.51 | | | | | 7.80e-13 | | | | | | |  | |
| 19708 | | precursor of fumarase fumarase | | | | | 27.72 | | | | 55.62 | | | | 1.00 | | | | | 0.00e+00 | | | | | | |  | |
| 30145 | | predicted protein | | | | | 41.56 | | | | 113.62 | | | | 1.45 | | | | | 8.67e-13 | | | | | | |  | |
| 42398 | | predicted protein | | | | | 40.84 | | | | 6.58 | | | | -2.63 | | | | | 3.33e-50 | | | | | | |  | |
| 46387 | | predicted protein | | | | | 9.46 | | | | 1.62 | | | | -2.55 | | | | | 4.13e-22 | | | | | | |  | |
| 50392 | | predicted protein | | | | | 2.76 | | | | 0.72 | | | | -1.94 | | | | | 2.37e-03 | | | | | | |  | |
| 46991 | | predicted protein | | | | | 550.42 | | | | 2457.21 | | | | 2.16 | | | | | 0.00e+00 | | | | | | |  | |
| 45428 | | predicted protein | | | | | 192.55 | | | | 1.96 | | | | -6.62 | | | | | 0.00e+00 | | | | | | |  | |
| 48393 | | predicted protein | | | | | 4.38 | | | | 1.32 | | | | -1.73 | | | | | 2.55e-09 | | | | | | |  | |
| 16069 | | predicted protein | | | | | 3.51 | | | | 0.73 | | | | -2.27 | | | | | 6.22e-07 | | | | | | |  | |
| 45557 | | predicted protein | | | | | 61.56 | | | | 54.33 | | | | -0.18 | | | | | 1.14e-01 | | | | | | |  | |
| 26290 | | predicted protein | | | | | 345.16 | | | | 677.39 | | | | 0.97 | | | | | 0.00e+00 | | | | | | |  | |
| 10356 | | succinate dehydrogenase cytochrome b subunit | | | | | 728.70 | | | | 1047.77 | | | | 0.52 | | | | | 0.00e+00 | | | | | | |  | |
| 41812 | | succinate dehydrogenase flavoprotein | | | | | 214.42 | | | | 283.68 | | | | 0.40 | | | | | 0.00e+00 | | | | | | |  | |
| 52539 | | succinate dehydrogenase iron sulfur protein | | | | | 210.72 | | | | 504.10 | | | | 1.26 | | | | | 1.86e-11 | | | | | | |  | |
| **f) Glycerolipid metabolism** | | | | | | | | | | | | | | | | | | | | | | | | |  | | | |
| 43099 | | | 1-acyl-sn-glycerol-3-phosphate acyltransferase | | | | 29.10 | | | 4.55 | | | | | -2.68 | | | 1.84e-54 | | | | | |  | | | | |
| 51608 | | | 2,5-diketo-D-gluconic acid reductase A-like protein | | | | 9.56 | | | 0.51 | | | | | -4.24 | | | 1.98e-13 | | | | | |  | | | | |
| 42962 | | | 2-phosphoglycerate dehydratase | | | | 16.07 | | | 6.78 | | | | | -1.24 | | | 7.15e-19 | | | | | |  | | | | |
| 9794 | | | diacylglycerol acyltransferase | | | | 10.78 | | | 7.22 | | | | | -0.58 | | | 1.06e-02 | | | | | |  | | | | |
| 54709 | | | glycerol-3-phosphate o-acyltransferase | | | | 86.93 | | | 23.17 | | | | | -1.91 | | | 1.45e-164 | | | | | |  | | | | |
| 50356 | | | glycosyl transferase, group 1 | | | | 1.67 | | | 0.56 | | | | | -1.59 | | | 6.97e-03 | | | | | |  | | | | |
| 14125 | | | monogalactosyldiacylglycerol synthase | | | | 137.24 | | | 15.19 | | | | | -3.18 | | | 2.63e-244 | | | | | |  | | | | |
| 40261 | | | phosphatidate phosphatase | | | | 26.49 | | | 33.02 | | | | | 0.32 | | | 2.50e-02 | | | | | |  | | | | |
| 8860 | | | phospholipid:diacylglycerol acyltransferase | | | | 323.97 | | | 613.93 | | | | | 0.92 | | | 2.06e-11 | | | | | |  | | | | |
| 43684 | | | predicted protein | | | | 288.92 | | | 35.29 | | | | | -3.03 | | | 0.00e+00 | | | | | |  | | | | |
| 42736 | | | predicted protein | | | | 51.26 | | | 12.02 | | | | | -2.09 | | | 3.43e-59 | | | | | |  | | | | |
| 30823 | | | predicted protein | | | | 11.10 | | | 2.46 | | | | | -2.18 | | | 1.53e-12 | | | | | |  | | | | |
| 14736 | | | predicted protein | | | | 8.17 | | | 1.53 | | | | | -2.41 | | | 9.42e-09 | | | | | |  | | | | |
| 48100 | | | predicted protein | | | | 3.12 | | | 2.02 | | | | | -0.63 | | | 1.52e-01 | | | | | |  | | | | |
| 3262 | | | predicted protein | | | | 1.85 | | | 12.02 | | | | | 2.70 | | | 2.47e-06 | | | | | |  | | | | |
| 42446 | | | predicted protein | | | | 17.41 | | | 8.49 | | | | | -1.04 | | | 7.02e-07 | | | | | |  | | | | |
| 50770 | | | predicted protein | | | | 74.25 | | | 16.47 | | | | | -2.17 | | | 3.58e-121 | | | | | |  | | | | |
| 50182 | | | predicted protein | | | | 19.54 | | | 4.81 | | | | | -2.02 | | | 5.22e-32 | | | | | |  | | | | |
| 12881 | | | predicted protein | | | | 13.01 | | | 0.36 | | | | | -5.20 | | | 1.67e-37 | | | | | |  | | | | |
| 42527 | | | predicted protein | | | | 6.76 | | | 1.36 | | | | | -2.31 | | | 2.08e-13 | | | | | |  | | | | |
| 14202 | | | predicted protein | | | | 36.54 | | | 31.65 | | | | | -0.21 | | | 1.23e-01 | | | | | |  | | | | |
| 47445 | | | predicted protein | | | | 7.93 | | | 1.51 | | | | | -2.39 | | | 3.21e-15 | | | | | |  | | | | |
| 43463 | | | predicted protein | | | | 5.32 | | | 1.19 | | | | | -2.15 | | | 3.19e-11 | | | | | |  | | | | |
| 41624 | | | predicted protein | | | | 2.25 | | | 0.13 | | | | | -4.11 | | | 1.99e-08 | | | | | |  | | | | |
| 43352 | | | predicted protein | | | | 104.32 | | | 69.28 | | | | | -0.59 | | | 1.45e-29 | | | | | |  | | | | |
| 34489 | | | predicted protein | | | | 178.81 | | | 209.18 | | | | | 0.23 | | | 7.33e-05 | | | | | |  | | | | |
| 41254 | | | predicted protein | | | | 2.71 | | | 0.41 | | | | | -2.73 | | | 5.32e-04 | | | | | |  | | | | |
| 31492 | | | predicted protein | | | | 0.97 | | | 2.91 | | | | | 1.59 | | | 3.69e-04 | | | | | |  | | | | |
| 39949 | | | predicted protein | | | | 5.14 | | | 1.68 | | | | | -1.61 | | | 3.59e-04 | | | | | |  | | | | |
| 9619 | | | predicted protein | | | | 74.35 | | | 13.15 | | | | | -2.50 | | | 8.30e-107 | | | | | |  | | | | |
| 54168 | | | predicted protein | | | | 9.50 | | | 1.63 | | | | | -2.54 | | | 1.28e-19 | | | | | |  | | | | |
| 42467 | | | predicted protein | | | | 368.00 | | | 1666.84 | | | | | 2.18 | | | 4.61e-11 | | | | | |  | | | | |
| 50372 | | | predicted protein | | | | 0.69 | | | 1.14 | | | | | 0.72 | | | 1.17e-01 | | | | | |  | | | | |
| 43116 | | | predicted protein | | | | 57.57 | | | 17.56 | | | | | -1.71 | | | 5.21e-97 | | | | | |  | | | | |
| 12884 | | | predicted protein | | | | 68.45 | | | 14.17 | | | | | -2.27 | | | 7.67e-90 | | | | | |  | | | | |
| 11390 | | | predicted protein | | | | 42.75 | | | 6.22 | | | | | -2.78 | | | 3.09e-68 | | | | | |  | | | | |
| 2215 | | | predicted protein | | | | 0.20 | | | 12.37 | | | | | 5.98 | | | 1.87e-04 | | | | | |  | | | | |
| 45551 | | | predicted protein | | | | 65.71 | | | 381.55 | | | | | 2.54 | | | 0.00e+00 | | | | | |  | | | | |
| 20460 | | | predicted protein | | | | 17.61 | | | 1.50 | | | | | -3.56 | | | 6.59e-49 | | | | | |  | | | | |
| 48799 | | | predicted protein | | | | 179.47 | | | 165.80 | | | | | -0.11 | | | 2.75e-04 | | | | | |  | | | | |
| 1971 | | | predicted protein | | | | 14.65 | | | 1.26 | | | | | -3.53 | | | 9.95e-37 | | | | | |  | | | | |
| 21201 | | | UDP-sulfoquinovose synthase, plastid precursor | | | | 101.08 | | | 13.10 | | | | | -2.95 | | | 2.29e-230 | | | | | |  | | | | |
| **g) Lipases** | | | | | | | | | | | | | | | | | | | | | | | | |  | | | |
| 42683 | | | | | 1-phosphatidylinositol-4,5- bisphosphate phosphodiesterase | | 15.61 | | | | | 6.28 | | -1.31 | | | 1.23e-20 | | | | | |  | | | | | |
| 49771 | | | | | 1-phosphatidylinositol-4,5- bisphosphate phosphodiesterase | | 0.68 | | | | | 1.62 | | 1.24 | | | 2.54e-04 | | | | | |  | | | | | |
| 34489 | | | | | acylglycerol lipase | | 178.81 | | | | | 209.18 | | 0.23 | | | 7.33e-05 | | | | | |  | | | | | |
| 41254 | | | | | acylglycerol lipase | | 2.71 | | | | | 0.41 | | -2.73 | | | 5.32e-04 | | | | | |  | | | | | |
| 43352 | | | | | acylglycerol lipase | | 104.32 | | | | | 69.28 | | -0.59 | | | 1.45e-29 | | | | | |  | | | | | |
| 44005 | | | | | carboxylic ester hydrolases | | 16.31 | | | | | 7.02 | | -1.22 | | | 7.20e-25 | | | | | |  | | | | | |
| 31492 | | | | | lipase | | 0.97 | | | | | 2.91 | | 1.59 | | | 3.69e-04 | | | | | |  | | | | | |
| 49702 | | | | | lysophospholipase | | 5.02 | | | | | 0.94 | | -2.41 | | | 2.23e-18 | | | | | |  | | | | | |
| 48445 | | | | | phospholipase C | | 6.50 | | | | | 1.57 | | -2.05 | | | 6.23e-10 | | | | | |  | | | | | |
| 41624 | | | | | triacylglycerol lipase | | 2.25 | | | | | 0.13 | | -4.11 | | | 1.99e-08 | | | | | |  | | | | | |
| 43463 | | | | | triacylglycerol lipase | | 5.32 | | | | | 1.19 | | -2.15 | | | 3.19e-11 | | | | | |  | | | | | |
| 47010 | | | | | triacylglycerol lipase | | 4.60 | | | | | 2.50 | | -0.88 | | | 4.34e-05 | | | | | |  | | | | | |
| 47445 | | | | | triacylglycerol lipase | | 7.93 | | | | | 1.51 | | -2.39 | | | 3.21e-15 | | | | | |  | | | | | |
| 37711 | | | | | triacylglycerol lipase activity | | 16.85 | | | | | 7.99 | | -1.08 | | | 1.37e-07 | | | | | |  | | | | | |
| 43593 | | | | | triacylglycerol lipase activity | | 21.32 | | | | | 74.84 | | 1.81 | | | 1.05e-12 | | | | | |  | | | | | |
| 44028 | | | | | triacylglycerol lipase activity | | 582.17 | | | | | 1800.15 | | 1.63 | | | 0.00e+00 | | | | | |  | | | | | |
| 44231 | | | | | triacylglycerol lipase activity | | 15.76 | | | | | 3.02 | | -2.38 | | | 6.12e-46 | | | | | |  | | | | | |
| 47614 | | | | | triacylglycerol lipase activity | | 4.98 | | | | | 1.76 | | -1.50 | | | 2.49e-10 | | | | | |  | | | | | |
| 47650 | | | | | triacylglycerol lipase activity | | 38.12 | | | | | 6.57 | | -2.54 | | | 4.53e-208 | | | | | |  | | | | | |
| 50397 | | | | | triacylglycerol lipase activity | | 10.47 | | | | | 2.74 | | -1.93 | | | 1.67e-25 | | | | | |  | | | | | |

RPKM stands for Reads Per Kilobase per Million mapped reads. Fold changes in transcripts are listed with delta values yielding FDRs <0.1%. Values presented are log_2_ ratios of expression (N deprivation/control). a) primary metabolism, b) nitrogen metabolism, c) photosynthesis, d) carbohydrate metabolism, e) TCA cycle, f) glycerolipid metabolism, g) lipases.
